# A *sex-ratio* Meiotic Drive System in *Drosophila simulans.* I: An Autosomal Suppressor

**DOI:** 10.1371/journal.pbio.0050292

**Published:** 2007-11-06

**Authors:** Yun Tao, John P Masly, Luciana Araripe, Yeyan Ke, Daniel L Hartl

**Affiliations:** 1 Department of Organismic and Evolutionary Biology, Harvard University, Cambridge, Massachusetts, United States of America; 2 Department of Biology, Emory University, Atlanta, Georgia, United States of America; 3 Department of Biology, University of Rochester, Rochester, New York, United States of America; Cornell University, United States of America

## Abstract

Sex ratio distortion (*sex-ratio* for short) has been reported in numerous species such as *Drosophila*, where distortion can readily be detected in experimental crosses, but the molecular mechanisms remain elusive. Here we characterize an autosomal *sex-ratio* suppressor from D. simulans that we designate as *not much yang* (*nmy,* polytene chromosome position 87F3). *Nmy* suppresses an X-linked *sex-ratio* distorter, contains a pair of near-perfect inverted repeats of 345 bp, and evidently originated through retrotransposition from the distorter itself. The suppression is likely mediated by sequence homology between the suppressor and distorter. The strength of *sex-ratio* is greatly enhanced by lower temperature. This temperature sensitivity was used to assign the *sex-ratio* etiology to the maturation process of the Y-bearing sperm, a hypothesis corroborated by both light microscope observations and ultrastructural studies. It has long been suggested that an X-linked *sex-ratio* distorter can evolve by exploiting loopholes in the meiotic machinery for its own transmission advantage, which may be offset by other changes in the genome that control the selfish distorter. Data obtained in this study help to understand this evolutionary mechanism in molecular detail and provide insight regarding its evolutionary impact on genomic architecture and speciation.

## Author Summary

Genetic conflicts among genes happen when their modes of transmission differ. Genes in the heterogametic (XY) sex can be grouped as X-linked, Y-linked, autosomal, or cytoplasmic. Sex ratio in the progeny greatly affects the transmission advantage of each of the four types of genes, with the optimal sex ratio for each type being respectively 100%, 0%, 50%, and 100% of females. Sex ratio can often be biased from the normal 50% by genes that distort the sex ratio toward their own optimal transmission. Here we report genetic and molecular characterization of an autosomal gene that functions as a suppressor of an X-linked *sex-ratio* distorter. Male mutants give rise to female-biased progeny. The cause of the distortion was assigned to a failure of the Y-bearing sperm to mature. The DNA sequence of the suppressor gives clues to understanding the *sex-ratio* meiotic drive at the molecular level. More generally, the genetic conflict over sex ratio may be important in determining the evolutionary dynamics and the architecture of eukaryotic genomes.

## Abstract 3

Autosomal genes defend faithful Mendelian segregation by suppressing sex-ratio distorters on the X chromosome. The molecular mechanisms of this kind of genomic policing have begun to be elucidated.

## Introduction

As a rule, most dioecious species produce equal numbers of male and female progeny because the rarer sex has a mating advantage. R. A. Fisher in 1930, as well as Carl Düsing in 1884 before him, framed arguments based on parental expenditure that mandates an equilibrium sex ratio of 50% female [1,2]. This so-called Fisher's principle is based on the premise that genes have maximum representation in future generations only if they are equally transmitted through both sexes [3]. However, sex ratios in nature sometimes show marked departures from 50%, and there are many ecological and genetic situations where the assumptions of Fisher's principle do not hold [4,5]. One prominent example is related to sex-linked genes of the heterogametic sex. Because of their unisexual transmission, mutations that distort their meiotic transmission—thus the sex ratio—in their own favor can gain selective advantage [6]. This type of sex ratio meiotic drive, also called *sex-ratio*, conflicts with selection for equal transmission of autosomal genes and thus represents a typical intragenomic conflict among genes when their transmission optima are not congruent.

A *sex*-*ratio* distorter can invade a population as long as its deleterious effects on viability and fertility are offset by the biased segregation [7]. Invasion by a *sex*-*ratio* distorter creates a genetic context promoting strong selection for sex-linked or autosomal suppressors that ameliorate the segregation distortion and/or its deleterious pleiotropic effects [8]. This type of recurrent intragenomic conflict generates a dynamic of genetic “attack” and “defense,” even in the absence of any external abiotic or biotic challenges [4,9,10]. As a result, genetic conflicts over sex ratio may be a key factor in driving the evolution of gametogenesis in the heterogametic sex, thereby leading to the nearly ubiquitous pattern of Haldane's rule observed in speciation [11–13]. Because the evolution of genetic suppression would make *sex-ratio* transient on an evolutionary time scale, the uncovering of suppressed *sex-ratio* mutations requires sophisticated crossing schemes, often between individuals from different populations or incipient species. Perhaps it is because *Drosophila* geneticists have often studied matings of this type that most of the known examples of *sex-ratio* are found in *Drosophila* [6], and new cases continue to be reported regularly [14–16].

At least three independent *sex-ratio* meiotic drive systems have been uncovered in the species D. simulans [15,17–20]. To facilitate discussion, we will assign each *sex-ratio* system a different name according to the location where the original stocks were collected or the research was carried out. In the most thoroughly analyzed case, which we will refer to as the Paris *sex-ratio*, at least two X-linked distorters have been mapped, and they are currently being characterized at the molecular level [19,21,22]. The Paris *sex-ratio* (*SR*) appears to have originated relatively recently, and evolutionary signatures of a recent selective sweep are evident [23]. Multiple suppressors on the autosomes and the Y chromosome have been reported [19,21,24]. Both the distorters and the suppressors are polymorphic across the worldwide distribution of D. simulans [25–28]. Cytogenetic studies show that most (92%–96%) of the Y chromosomes in *SR* males do not undergo normal disjunction during meiosis II. As a consequence, the Y chromosome is often fragmented and lost. Sperm without a Y chromosome do participate in fertilization, and account for the 15%–30% of sterile F_1_ progeny males observed [29]; however most of the Y-bearing spermatids fail to mature in spermiogenesis [30]. The second *sex-ratio* system, which we call the Durham *sex*-*ratio*, was uncovered by introgressing regions of the third chromosome from D. mauritiana into *D. simulans.* The gene responsible for the suppression was named *too much yin* (*tmy*) [15]. An intriguing observation is that among about 20 hybrid male sterility loci on the third chromosome between these two species, *tmy* also had the strongest sterilizing effect on hybrid males. This observation provides a direct link between meiotic drive and interspecific hybrid male sterility [15]. The third *sex-ratio* system, which we call the Winters *sex-ratio*, was first revealed in interspecific hybrids between D. simulans and D. sechellia, using a D. simulans stock collected in Winters, California [20]. The gene responsible for revealing the *sex-ratio* is autosomal recessive. It was thought to have been introgressed from D. sechellia into a largely D. simulans genetic background and to have replaced the dominant suppressing D. simulans allele; however, two alternative explanations could not be ruled out. In the first alternative, a cryptic meiotic drive system from D. sechellia may have been introgressed into the D. simulans background and became reactivated. In the second alternative, a *sex-ratio* system may have been created de novo by an interaction between D. simulans and D. sechellia genes, even though these genes are irrelevant to sex ratio control in their native genomes [20].

Two additional *sex-ratio* cases have been reported in D. simulans [17,18]. For a case discovered in Brazil, an autosomal recessive mutation was implicated [18]. Similarly, in flies collected in California in 1959 [17], a recessive mutation (*sxr*) was mapped on the third chromosome. Unfortunately, the stocks of these two cases are no longer available and hence their relationship to the above three systems cannot be ascertained.

Given the frequent observations of *sex-ratio* and its potential biological significance, it is surprising that no single gene has as yet been identified for any *sex*-*ratio* system. Two difficulties prevent a molecular genetic analysis. First, many *sex-ratio* cases have been found in the genus *Drosophila* but not yet in natural populations of the model species D. melanogaster. Second, *sex-ratio* genes are often associated with inversions, making even conventional genetic mapping difficult. In our study we took advantage of the genomic and genetic resources developed in recent years for *Drosophila* to characterize the Winters *sex-ratio* system at the molecular level. Evidence for the existence of the other two independent *sex-ratio* meiotic drive systems in D. simulans will also be elaborated. The study of *sex-ratio* systems may help us gain new insights into mechanisms of speciation, the maintenance of Mendelian segregation, and the evolutionary principles of genome organization.

## Results

### A Recessive Mutation of the *sex-ratio* Suppressor on the Third Chromosome

**Figure 1:**
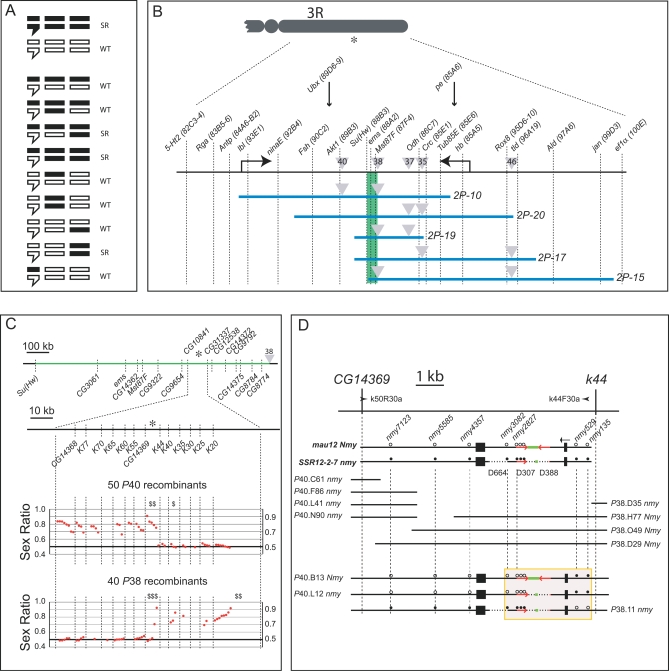
Positional Cloning of *not much yang*, a Recessive Autosomal *sex-ratio* Suppressor on the Third Chromosome (A) A recessive gene on the third chromosome was implicated for uncovering *sex-ratio*. Through various crossing schemes (see Text S1 for details), chromosomal combinations from *sex-ratio* (black) and wild-type (white) strains were tested for *sex-ratio*. In each genotype, bars from left to right represent the X, the second, and the third chromosome, respectively. The bar with a hook represents the Y chromosome. The fourth chromosome was not followed. SR: *sex-ratio*; WT: wild type (not *sex-ratio*). (B) A major gene was mapped between *P*40 and *P*38 on the right arm of the third chromosome (3R). Shown here are the 2-*P* lines used in the mapping, with the blue bars representing the D. mauritiana portion of the introgression lines and the gray triangles representing the *P*[*w* ^+^] inserts (see Figure S6 for details). The two bent arrows represent the 84F-93F inversion in D. simulans complex as compared to D. melanogaster. Dotted lines represent ASO markers. The target gene (*) falls within the green region that was subsequently mapped in more detail. (C) Fine mapping at 1-kb resolution. Fifty *P*40 and 40 *P*38 recombinants were found with their crossovers falling in the *CG10841–CG31337* region. The positions of these crossovers were delimited with 13 ASO markers. The sex ratio (proportion of female) for each recombinant is shown here in rank order within each ASO interval. Significant among-recombinant variation in sex ratio is indicated (one-way ANOVA: $, *p* < 5%; $$, *p* < 1%, and $$$, *p* < 0.1%). The target major gene (*) is mapped to the 7-kb interval of *CG14369–K44*. (D) Two insertions/deletions are responsible for the *nmy* phenotype. The parental sequences from *D. mauritiana w* (*mau12 Nmy*) and *D. simulans SSR12-2-7 nmy* were amplified by long PCR using the primers k50R30a and k44F30a (arrow heads, see Text S2 for sequences). Empty and filled circles represent a subset of the informative substitutions. The filled rectangles represent the coding sequence of *CG14370*, which is a single exon gene in D. melanogaster. The sequences between the two filled rectangles, 2,791 bp in *mau12 Nmy* and 1,427 bp in *SSR12-2-7 nmy*, are missing in D. melanogaster. The arrow represents the transcription orientation. Dashed lines represent, from left to right, three large deletions of 664, 307, and 388 bp in *SSR12-2-7 nmy*, respectively. The red arrows stand for inverted repeats (IR) of 380 bp in *mau12 Nmy*, and a 345-bp homolog in the *SSR12-2-7 nmy* allele. The green lines represent sequences between the IRs. The last informative 11 recombinants were genotyped with seven ASO probes (*nmy135 – nmy7123*). Eight of the 11 recombinants (*P*40.C61 to *P*38.D29) are shown with their D. mauritiana portions (bars). The three most informative recombinants were sequenced with their crossovers precisely determined. The only difference between *P*40.B13 *Nmy* and *P*40.L12 *nmy* is the last two insertions/deletions (shaded box).

We first mapped a recessive gene responsible for uncovering the Winters *sex-ratio* to the vicinity of the locus *pe* (polytene chromosome position 85A6) on the third chromosome through various crosses by using stocks from a previous study, where the Winters *sex-ratio* was revealed [20] (Text S1; Figures S1–S5). We reasoned that the recessive allele is likely a loss-of-function mutation of a dominant *sex-ratio* suppressor, rather than corresponding to the distorter itself, because a *sex-ratio* distorter has no transmission advantage unless it is sex-linked (Figure 1A). A *sex-ratio* line *SSR12-2-7* was constructed by extracting the third chromosome from the stock *SSR12* into a pure D. simulans background (Figure S2). This stock was used throughout this study for genetic and phenotypic analyses.

Further mapping of the *sex-ratio* suppressor was made possible through a 2-*P* mapping scheme [15], where the *P*[*w* ^+^]-tagged D. mauritiana introgression stocks were constructed for other purposes (mapping hybrid male sterility between D. mauritiana and D. simulans) [31]. The *P* inserts here conveniently functioned as semi-dominant markers (Figure S6). Their positions and the introgression lines with two *P* inserts (2-*P* lines) in the *pe* region are shown in Figure 1B. The results of the 2-*P* mapping clearly indicate that the target gene must be localized to a ∼2,700-kb interval between *P*40 and *P*38 (Figure S6). With allele-specific oligonucleotide (ASO) markers (Table S2), we genotyped 129 recombinants generated from the 2*P-10* line to narrow down the target to an interval of 88 kb between *CG10841* and *CG31337* (Figure 1C). There are no obvious candidate genes for the *sex-ratio* suppressor in this region.

To pinpoint the target gene more precisely, we screened an additional 3,100 recombinants within the *P*40 – *P*38 interval with 40 *P*38 and 50 *P*40 recombinants falling within the 88-kb interval between *CG10841* and *CG31337* (Figure 1C). Analysis of 79 of these recombinants narrowed the target to the 7-kb interval between *CG14369* and *K44* region (Figure 1C**)**. In such a small region, it is unlikely that more than one gene is responsible for uncovering the Winters *sex-ratio.* We named this gene *not much yang* (*nmy*). We use the symbol *Nmy* to denote the dominant suppressing allele in D. simulans and D. mauritiana. The recessive loss-of-function allele that allows the *sex-ratio* phenotype to be expressed is denoted *nmy*.

We sequenced the parental chromosomes from *D. mauritiana mau12* (*Nmy*) and *D. simulans SSR12-2-7* (*nmy*) across the *CG14369–K44* interval (Figure 1D). From this region, sequences homologous to *CG14370* of D. melanogaster were recognized. Insertions of 2,791 bp and 1,427 bp were found in the coding region of *CG14370* in *mau12* (*Nmy*) and *SSR12-2-7* (*nmy*), respectively. Notably, a pair of inverted repeats of 380 bp was found in the 2.8-kb *Nmy* insert, and three large deletions were found in the 1.4-kb *nmy* insert relative to the 2.8-kb *Nmy* insert. Because of these deletions, only one repeat remains in the 1.4-kb *nmy* insert (Figure 1D). The target region was further narrowed down to the 2.3-kb interval of *nmy529–nmy2827* by genotyping the last 11 recombinants with seven additional ASO probes that mark the region from *nmy135* to *nmy7123* (Figure 1D, Table S3). Sequencing showed that the only difference between *P*40.B13 *Nmy* and *P*40.L12 *nmy* is in the two deletions D307 and D388 (Figure 1D). We conclude that the wild-type function of *Nmy* as a *sex-ratio* suppressor will be lost if the pair of inverted repeats in the *Nmy* insert are not intact. Hereafter, we use brackets to denote the species and length of the insert for the *Nmy* allele. For example, *Nmy*[*mau2791*] and *nmy*[*sim1427*] are used to represent the alleles as well as the inserts found in *mau12* and *SSR12-2-7*, respectively.

### Gene Structure and Origin of *Nmy*

**Figure 2:**
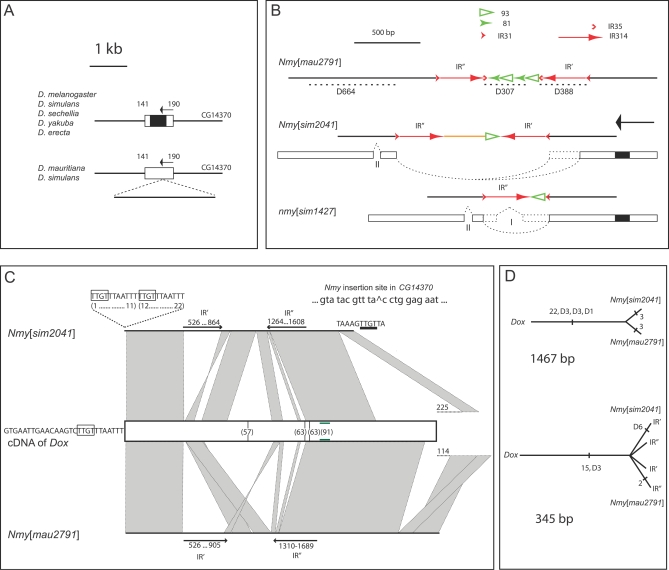
The Molecular Structure and Evolution of *Nmy* (A) An incomplete survey of *Nmy/CG14370* alleles found in species of the D. melanogaster subgroup. *CG14370* is a single-exon gene in most species, but various inserts are found in D. simulans and D. mauritiana that represent alleles of *Nmy.* Arrow: transcription orientation; rectangle: transcripts; filled: coding sequence; empty: untranslated region. The status of translation in D. simulans and D. mauritiana is unclear. (B) Comparison of the three *Nmy* inserts found in D. mauritiana and D. simulans. For each allele, solid lines indicate the genomic sequence with various sequence components marked with symbols (e.g., an empty triangle for a 93-bp element). For *Nmy*[*sim2041*] and *nmy*[*sim1427*]. Partial transcripts of various lengths are shown as rectangles (filled: coding sequence as in *CG14370* but with earlier termination; dotted: alternative transcripts). Black arrow indicates transcription orientation. Two likely alternatively spliced introns (I and II) were identified. Possible spurious reverse-transcription products were also indicated (curved dotted line, see Text S3 for more details). (C) Origin of *Nmy* inserts by retrotransposition. The cDNA of *Dox*, *Nmy*[*sim2041*], and *Nmy*[*mau2791*] are compared and the paralogous regions are highlighted (parallelogram; its twisted form for reverse orientation). The positions of three introns of 57, 63, and 63 bp (vertical broken bar with intron size) and an alternative intron of 91 bp (horizontal green bar) from *Dox* are shown. Nucleotides at the 5′ ends of the two *Nmy* alleles and the cDNA of *Dox* are shown; also shown are the 3′ end of *Nmy*[*sim2041*]. Eleven nucleotides, TTGTTTAATTT, near the 3′ end of the *Dox* transcription were duplicated during the retrotransposition event. The insertion site within *CG14370* for the retrotranspostion is also shown. The dinucleotide, TA, before the insertion target (^) was also duplicated, but the nearby tetranucleotides TTGT (underlined) might not be related to the other TTGT (framed). The 3′ end sequences from both *Nmy* alleles fail to match to the extant cDNA of *Dox*, but the sequence does match to the genomic regions 114 and 225 bp upstream of *Dox*, respectively (dotted lines with the number of 114 or 225). The left-hand inverted repeat IR' was most likely generated as a secondary duplication after the retrotransposition event. (D) Both *Nmy*[*sim2041*] and *Nmy*[*mau2791*] evolved from a common retrotransposed sequence of *Dox*. Upper: Homologous sequences of 1,467 bp among *Dox*, *Nmy*[*sim2041*], and *Nmy*[mau2791] were used to construct a star phylogeny with the number of single nucleotide substitutions as well as the three insertions/deletions of 1–3 nucleotides (D3, D3, and D1) mapped on the branches. Lower: Homologous sequences of 345 bp among the two inverted repeats of *Nmy*[*sim2041*], *Nmy*[mau2791], and *Dox* were used to construct another star phylogeny. The four inverted repeat sequences are essentially identical except for a 6-bp deletion (D6) in IR' of *Nmy*[*sim2041*], and two nucleotide substitutions in IR'' of *Nmy*[mau2791].

We compared the *CG14370/Nmy* region from several species of the D. melanogaster subgroup. The *Nmy* inserts are found in D. simulans (four strains) and D. mauritiana (one strain) but not in the other species (one strain each) (Figure 2A). Importantly, we found no *Nmy* insert in the D. sechellia strain (3588) but two inserts (*Nmy*[*sim2041*] and *nmy*[*sim1427*]) in the D. simulans strain (*sim2*). These two strains were used in constructing the D. simulans × D. sechellia recombinant inbred lines where the Winters *sex-ratio* was first observed [20]. Evidently, the allele *nmy*[*sim1427*] does not come from D. sechellia. Rather, it is still segregating in the *sim2* strain with a frequency of 6.1% (*n* = 294). There is an intact inverted-repeat structure within the allele *Nmy*[*sim2041*] that has full function as a *sex-ratio* suppressor (unpublished data). In addition, a null allele of *nmy* (i.e., *CG14370* without any insert) was also found in a local Massachusetts population of D. simulans (unpublished data). Taken together, the evidence indicates that *Nmy* is a gain-of-function mutation that suppresses the Winters *sex-ratio* distorter.

The three *Nmy* inserts are compared in Figure 2B. The length of a single inverted repeat in *Nmy*[*mau2791*] is 35 bp longer than that in *Nmy*[*sim2041*]. The sequences between the inverted repeats in these two *Nmy* alleles are not homologous, except for a 93-bp element found in reverse orientation. There is a 664-bp fragment (D664 in Figure 2B) in *Nmy*[*mau2791*] that is absent in the two D. simulans alleles. The allele *nmy*[*sim1427*] was derived from *Nmy*[*sim2041*] by the loss of one inverted repeat and the loss of most of the sequence located between the inverted repeats, except for the 93-bp element in reverse orientation. This observation again suggests that the inverted-repeat structure is essential for the *sex-ratio* suppression function. *Nmy* does not appear to be a conventional gene (Figure 2B). Both ends of the transcripts across the *Nmy*[*sim2041*] or *nmy*[*sim1427*] region were determined by 5′ and 3′ rapid amplification of cDNA ends (RACE) and they are identical to those of *CG14370* found in D. melanogaster. The full length of the mRNA from *nmy*[*sim1427*] was determined, and two alternately spliced introns (I, II) were identified (Figure 2B). We failed, however, to obtain the internal sequences of mRNA from *Nmy*[*sim2041*], because the reverse transcription was evidently stalled, most likely by a stem-loop secondary structure formed between the inverted repeats (see Text S3). The coding potential is limited for transcripts from both *nmy*[*sim1427*] and *Nmy*[*sim2041*] because of the premature termination of the *CG14370* open reading frame (ORF) (Figure 2B). Although we cannot rule out the existence of other functional ORFs in the *Nmy*[*sim2041*] transcripts, it seems more plausible that the stem-loop structure is functional and essential as a suppressor. We predict that small interfering RNAs (siRNAs) generated from this stem-loop structure would contain the information that is necessary for the specificity of suppression.

Further evidence for a possible siRNA mechanism comes from the mapping and cloning of a *sex-ratio* distorter on the X chromosome (*Dox*). *Dox* is a novel gene that is responsible for the Winters *sex-ratio* distortion, whereas *Nmy* suppresses *Dox* and results in a normal sex ratio [32]. Sequence comparisons strongly suggest that *Nmy* originated from part of *Dox* through retrotransposition (Figure 2C). The retrotransposition event is evident by the following observations. First, there is a tandem duplication of the dinucleotide (TA) at the insertion site within *CG14370*, and 11 base pairs (TTGTTTAATTT) that are proximal to the 5′ end of the *Dox* cDNA are also duplicated in the 5′ end of the *Nmy* insert. These two duplications are the telltale signs of a retrotransposition event through a target-primed reverse transcription mechanism, which are possibly catalyzed by some non–long terminal repeat type retrotransposon [33–35]. Second, the three introns of *Dox*, with lengths of 57, 63, and 63 bp, respectively, are not found in *Nmy*. On the other hand, it is unclear whether the precursor mRNA in the retrotransposition event was a bona fide transcript of *Dox*, because the 3′ end of the *Nmy* inserts matches to genomic region 100–200 bp upstream from *Dox*. There are several explanations for this discrepancy. First, the 5′ end of an alternative *Dox* transcript may have been missed in our 5′ RACE experiments. Second, the evolutionary precursor of the current *Dox* might have a longer transcript. Third, the *Nmy* insert may derive from a longer and aberrant transcript from *Dox*.

Sequence comparisons support further inferences about the molecular evolution of the *Nmy* gene. Other than the left-most inverted repeat (IR' in Figure 2C), sequences of *Nmy* and the cDNA from *Dox* are largely colinear, implying that IR' originated as a secondary duplication after the retrotransposition. Many nucleotide substitutions are shared by *Nmy*[*sim2041*] and *Nmy*[*mau2791*], although each also has its unique substitutes. This observation suggests that these two alleles shared a common ancestor before the split between D. simulans and D. mauritiana. Even though there are some gross sequence changes along the two *Nmy* lineages, their homologous sites have only minor differences (3/1,467bp) relative to their divergence from the *Dox* sequence (Figure 2D). Furthermore, the inverted repeats from all alleles are virtually identical, again suggesting the functional importance of the stem-loop secondary structure (Figure 2D).

### Etiology of the Winters *sex-ratio*: Abnormal Spermatogenesis

Distorted sex ratio can be caused by a number of mechanisms, including male-specific lethality, phenotypic sex reversal, or a deficiency of functional Y-bearing sperm. We inferred abnormalities in spermatogenesis from a cross of *sex-ratio* males with *C(1)RM y w* females, where the Y-bearing sperm are transmitted to female progeny. Progeny from this cross show a male biased sex ratio, implying a deficiency of functional Y-bearing sperm from *sex-ratio* males (Table 1). In another cross of *sex-ratio* males to females carrying the X-linked marker *forked* (*f*), no *f* female progeny were ever observed, ruling out the feminization of XY males to phenotypic females as the cause of the female-biased sex ratio (Table 1). Additionally, survival rates from egg to adult in the progeny of *sex-ratio* males are not different from controls, confirming earlier suggestion that male-specific lethality is not the cause of *sex-ratio* (Table S4) [20].

**Table 1:**
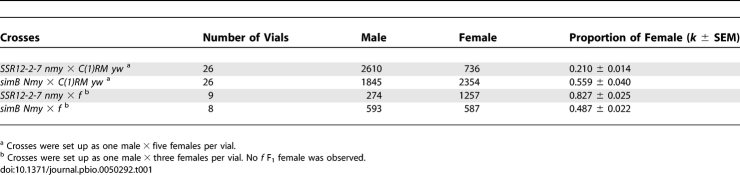
*Sex-ratio* Is Not Caused by a Zygotic Problem

**Figure 3:**
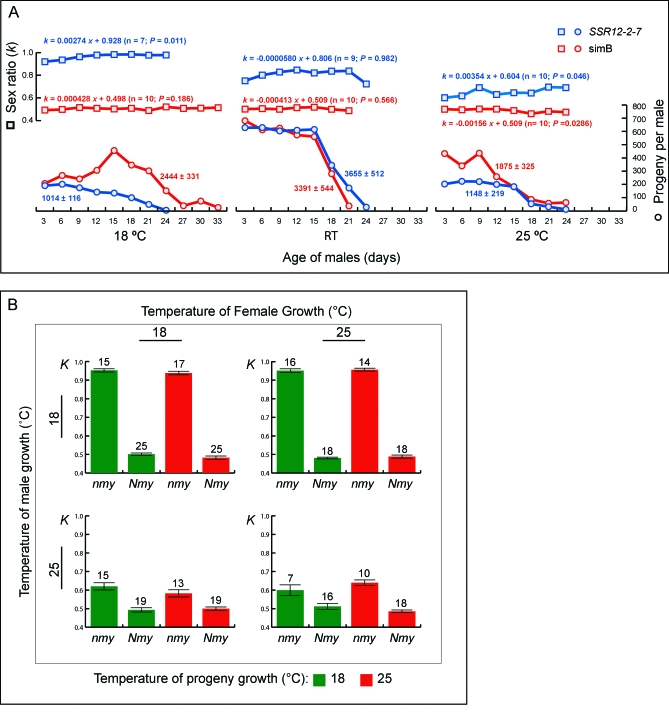
The Expression of s*ex-ratio* Is Temperature Sensitive during Spermatogenesis (A) The expression of *sex-ratio* is temperature sensitive. The sex ratio and size of progeny of *SSR12-2-7 nmy* or *simB Nmy* (control) males at various ages are shown. “Sperm exhaustion” experiments were done at three temperatures: 18 (± 0.5)°C, room temperature (22 ± 1.0°C), and 25 (± 0.5)°C. Every 3 d, single *SSR12-2-7 nmy* or *simB Nmy* males were provided with three *w; e* virgin females, and the mated *w; e* females were transferred to fresh vials. The procedure was repeated until the females were no longer fertile. In this fashion, progeny number (mean ± SEM per male) serves as a proxy for the number of functional sperm. The overall sex ratio (*k*) trend with age (*x*) is also shown (*k* = *ax* + *b*; *n* = males tested; *p* = significance test for the hypothesis *a =* 0). A significant age effect on *sex-ratio* was detected at all three temperatures (*k* = 0.00383*x* + 0.771; *p* = 0.046 for *SSR12-2-7 nmy* at room temperature if the last data point is excluded). The expressivity of *sex-ratio* is inversely correlated with the temperature. *SSR12-2-7 nmy* males were as fertile as *simB* at room tempreature and 25 °C (*t*-test, *p* = 0.729 and 0.08, respectively), but they were significantly lower in fertility at 18°C (*p* = 0.002). (B) The temperature sensitivity of *sex-ratio* is restricted to spermatogenesis. In this “temperature combination” experiment, pairs of 2-d-old virgin males (*SSR12-2-7 nmy* or *simB Nmy*) and females (*w; e*), reared at either 18 °C or 25 °C, were monitored for copulation up to 8 h at room temperature (22 ± 1 °C). Immediately after copulation, the male was aspirated away while the female was cultured at either 18 °C or 25 °C. These mated females were transferred to fresh vials every 3 d until the females became sterile. Seven to 25 males were tested for each of the 16 combinations of factors (genotype and temperature). The results (*sex-ratio* ± SEM) are shown as columns, and error bars with the sample sizes are also indicated. A generalized linear model was used for analysis of variance (SAS PROC GENMOD). Only the temperature at which males were reared had a significant effect on the observed *sex-ratio* (*p* < 0.0001), while the other two temperatures at which the females and the progeny were reared have no effect (*p* = 0.5703 and 0.3021, respectively).

A lack of functional Y-bearing sperm could be caused by failure in development, motility, or ability to fertilize. To distinguish among these possibilities, we exploited the temperature sensitivity of the Winters *sex-ratio*. In particular, at 18 °C, the sex ratio in progeny of *nmy* males can reach as high as 93%, whereas at 25 °C, the sex ratio decreases to about 60% (Figure 3A). The age of the males has a much smaller but also significant effect on the sex ratio. In experiments carried out in such a way that each functional sperm was maximized in its chance of fertilization, we estimated that a *nmy* male produces about as many sperm as a *simB* male does at room temperature or 25 °C, but produces less than half as many functional sperm at 18 °C (Figure 3A). To assess whether any reproductive process taking place in females contributes to the sex ratio distortion, we carried out an experiment in which *Nmy* and *nmy* males and females reared at two temperatures (18 °C and 25 °C) were mated, and the F_1_ progeny were reared separately at these two temperatures (Figure 3B). The resulting sex ratio depended only on the temperature at which the parental males were reared, suggesting that spermatogenesis, not sperm competition and/or fertilization, is the stage where the etiology of the *sex-ratio* happens.

### Cytological Evidence for Failure of the Y-Bearing Sperm Maturation

**Figure 4:**
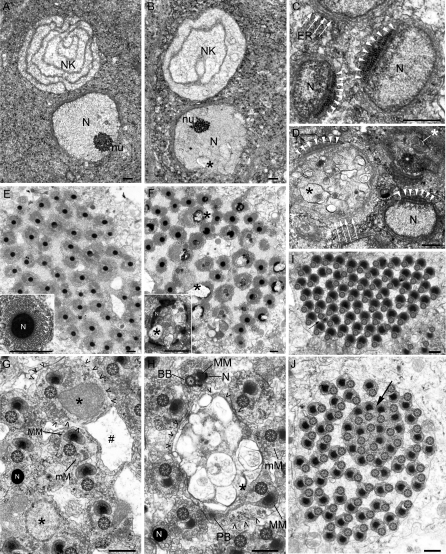
Abnormal Nuclear Transformation of the Y-Bearing Sperm Ultrastructural studies of sperm nuclear transformation and individualization in wild-type *simB Nmy* males (A, C, E, G, I) as compared with *SSR12-2-7 nmy* males (B, D, F, H, J) at 16 °C. All scale bars represent 500 nm. (A and B) Onion stage. The earliest abnormalities are detected as nucleoplasmic vacuoles (*) at the onion stage in the nuclei (N) of *nmy* (B) but not of *Nmy* (A) males. The grainy protein body, likely the nucleolus (nu) and the spherical nebenkern (NK), appear to be normal. (C and D) Elongation period. Normally, the nucleoplasm (N) is homogenous, and several rows of microtubules are aligned along the fenestrated portion of the nuclear membrane (arrowheads). A sheath of endoplasmic reticulum (ER) surrounds the nucleus (arrow). In *nmy* males, some nuclei at this stage have very pronounced nucleoplasmic vacuoles (*), as if the elimination of excess nucleoplasm through the fenestrated side has failed, although the alignment of microtubules is apparently normal (arrowheads, D). On the other hand, some nuclei appear normal, and show a reduced volume compared to the abnormal nuclei (N in D), suggesting some nucleoplasm has already been eliminated. (E and F) Post-elongation period. By the end of this period, the chromatin has been homogeneously condensed and most of the nuclear envelope has been eliminated (E). Meanwhile, arrays of microtubules (arrowheads in lower left insert) have become rigidly organized around the nuclei. However, the nuclear condensation process has been disrupted in many *nmy* nuclei, in which the nucleoplasmic vacuoles apparently block the condensation progress (* for examples shown in F). Note that nuclear condensation is always accompanied by apposed rows of microtubules (arrowheads in lower left insert of F), lack of which seems to correspond to failed chromatin condensation and the subsequent rupture of nuclear envelope nearby. (G–J) Individualization process. A cystic bulge is initiated at the head region and traverses through the entire length of the spermatid cyst. During this process, excess nucleoplasm, nuclear envelope, and syncytial bridges between spermatids are squeezed away into the waste bag (G). In a cross section through the cystic bulge near the head region, fenestrated (*), and nonfenestrated (#) nuclear envelopes are seen in the process of elimination. One axonemal complex is marked with “v”. For the nuclear head (N in G) that the cystic bulge has already passed, note the absence of surrounding microtubules. (I) In this cross section through the tail region after individualization, 62 spermatid tails are each invested in its own membrane and well separated. One abnormal tail is also seen (arrow). The other three of the 64 tails may have already been eliminated into the waste bag. (H) Nucleoplasmic vacuoles (*) within an abnormal spermatid are squeezed into the tail region, apparently causing physical difficulty for the cystic bulge to pass through. This may explain why individualization cannot proceed through for a cluster of 13 tails (arrow in J), while the other 46 tails appear normal. The other five of the 64 tails were probably eliminated into the waste bag. Also shown in (G) and (H) are some notable structural components within an axonemal complex including basal body (BB), major mitochondrial derivative (MM), minor mitochondrial derivative (mM), and paracrystalline body (PB).

An ultrastructural study of spermatogenesis from *Nmy* and *nmy* males further narrowed the etiology of *sex-ratio* to abnormal spermiogenesis. Normal spermiogenesis follows an elaborate program of nuclear elongation, microtubule assembly and depolymerization, chromatin condensation, elimination of excess nuclear envelope and neoplasm, and individualization at the final stage [36–38]. During this process, a vast majority (>99%) of the nuclear content is eliminated as waste, and the microtubules perform essential cytoskeletal and transport functions [38]. Abnormalities in various stages of spermiogenesis in *nmy* males are evident (Figure 4). In early spermiogenesis, the individual mitochondria aggregate and fuse to form two giant interleaved structures (the onion stage, or nebenkern). Normally, the nuclei appear to be filled with a homogeneously fine granulofibrillar material, punctuated with occasional coarse granules and a prominent protein body [39]. However, the mutant spermatid nuclei start to accumulate nucleoplasmic vacuoles at the late onion stage. This is the earliest morphological abnormality that might be associated with malfunctions in spermiogenesis (Figure 4A and 4B).

In the subsequent elongation period, the nuclear envelope has already been demarcated into two well-defined areas, fenestrated and nonfenestrated. In the early stage of elongation, the fenestrated portion of the nuclear envelope is apposed to rows of microtubules running parallel to the elongating axis (Figure 4C). In the following periods, the microtubules migrate from the fenestrated to the nonfenestrated areas, at the same time that chromatin condensation begins on the nonfenestrated side. Along with the continuing nuclear condensation, much of the nuclear envelope and excess nucleoplasm are eliminated. The end product is a highly packed lanceolate nucleus, which is only about 1/200 of the original size. It is believed that the microtubules play essential roles in the process of chromatin condensation by performing transport and support functions [40] (Figure 4E). In *nmy* genotypes, some spermatid nuclei develop nucleoplasmic vacuoles that become very large in the early elongation stage. Because the rows of microtubules appear to be normal (Figure 4D), the failed transport across the nuclear envelope is not likely caused by microtubule malfunctions. In other words, it is more likely a failed process within the nuclei (e.g., chromatin packaging) that underlies the formation of the nucleoplasmic vacuoles. Later in the post-elongation period, these vacuoles may cause rupture of the nuclear envelope when forces generated from the microtubule arrays as well as from chromatin condensation exert a strong pressure on it (Figure 4F).

As an apparent consequence of the failed chromatin condensation, the sperm individualization process in *nmy* males is also perturbed. Normally, the individualization complex (IC) is initiated at the head region of the spermatid bundle and traverses along the entire length of the bundle. Concomitant with the passing of the IC, excess nucleoplasm, nuclear envelope, syncytial bridges, and degenerated spermatids are stripped off and collected in the waste bag. Each individual spermatid becomes invested in its own membrane [36] (Figure 4G and 4I). In *nmy* males, the nucleoplasmic vacuoles are prominently present in the IC, likely blocking its passage through the tails (Figure 4H). These abnormal tails remain in the syncytium, while the rest of the tails are individualized and separated (Figure 4J).

Counts of abnormal and normal nuclei or tails in transmission electron microscopy (TEM) micrographs from testes developed at 16 °C or 26 °C are shown in Table 2. During sectioning, the grids were prepared from sections at least 10 μm apart to avoid sampling the same nuclei for imaging, whereas only one grid from each testis was used for the tail images. The average number of heads per bundle observed was much lower than the possible 64, in part because perfectly perpendicular sections through the heads of a spermatid bundle are rare. It is however noteworthy that the average number of heads (mean ± standard error of the mean [SEM] = 19.1 ± 1.0 per bundle, *n* = 80) from 16 °C *nmy* male is significantly fewer than that from control (27.4 ± 2.0, *n* = 51) (*t*-test, *p* < 0.001), suggesting the bundles in *nmy* males are less tightly packed in the head region because of the many big heads inflated by the large nucleoplasmic vacuoles (Figure 4D). For *nmy* males raised at 16 °C, neither the percentages of abnormal heads (25.2% ± 1.4%, *n* = 80) nor that of tails (30.6% ± 3.1%, *n* = 39) is sufficient to account for the biased sex ratio of 97.0%, because the maximum sex ratio would be 71.9% and 66.5%, respectively, assuming that all abnormal sperm are Y-bearing and the maximum sex ratio being calculated as 0.5 × (abnormal + normal)/normal. This discrepancy suggests that many Y-carrying spermatids with normal appearance in TEM were nonfunctional. For *nmy* males raised at 26 °C, there were very few abnormal heads and tails observed, consistent with the slight bias in sex ratio.

**Table 2:**
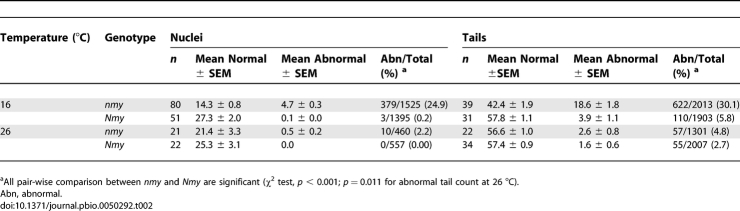
Spermatid Dimorphism Observed through TEM

**Figure 5:**
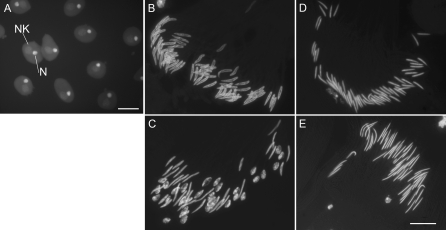
Abnormal Transformation of the Y-Bearing Sperm Detected by DAPI Staining (A) Onion stage (*SSR12-2-7 nmy*, 16 °C) similar to that in Figure 4A and 4B. Abnormal nucleoplasmic vacuoles are not visible under the fluorescence microscope. Nucleus (N) and nebenkern (NK) are indicated. Scale bar: 20 μm. (B and C) Spermatids of *SSR12-2-7 nmy* have strong dimorphism in nuclear transformation at 16 °C (C) as compared with *simB Nmy* (B). (D and E) The nuclear dimorphism of *SSR12-2-7 nmy* disappears at 26 °C (E) as compared to *simB Nmy* (D). Scale bar for B-E: 10 μm.

Under the fluorescence light microscope, the earliest sign of abnormal nuclear transformation can be detected in the elongation stage but not in the onion stage (Figure 5A–5C). After slight fixation and 4',6-diamidino-2-phenylindole (DAPI) staining, it is very easy to spread spermatid heads within a bundle. The dimorphism in nuclear transformation becomes very clear for *nmy* males developed at 16 °C but is attenuated with increasing temperature. Reared at 26 °C, *nmy* males have hardly any abnormal nuclei (Figure 5B–5E and Table 3). The correlation between predicted sex ratio and the sex ratio observed is highly significant (*r* ^2^ = 0.931, *p* = 0.0078). The high correlation implies that most if not all of the abnormally developed spermatids are Y-bearing.

**Table 3:**
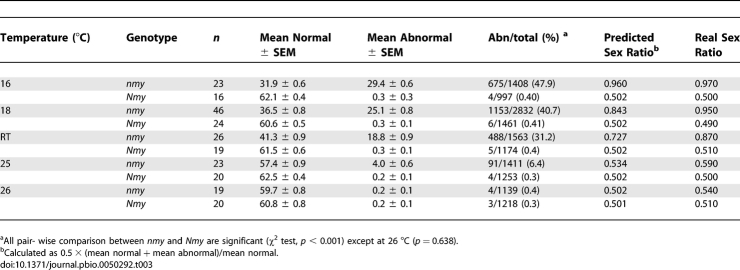
Spermatid Dimorphism Observed after DAPI Staining

### Failure of the Y-bearing Sperm Maturation: Evidence from the Temperature Shift Experiments

**Figure 6:**
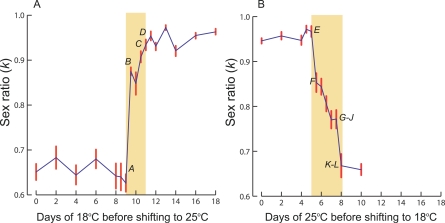
Temperature-Shift Experiment At various time points during development from eggs to adults, vials of *SSR12-2-7* (*nmy*) flies were shifted from 18 °C to 25 °C (A) or from 25 °C to 18 °C (B). Seven to 32 males eclosed from each of these vials were tested for *sex-ratio*, and the results (sex ratio and error bar = SEM) are shown at the time points of temperature shift. The stages of spermatogenesis at critical points (A–L) were examined cytologically (see Figure 7 for the corresponding images).

**Figure 7:**
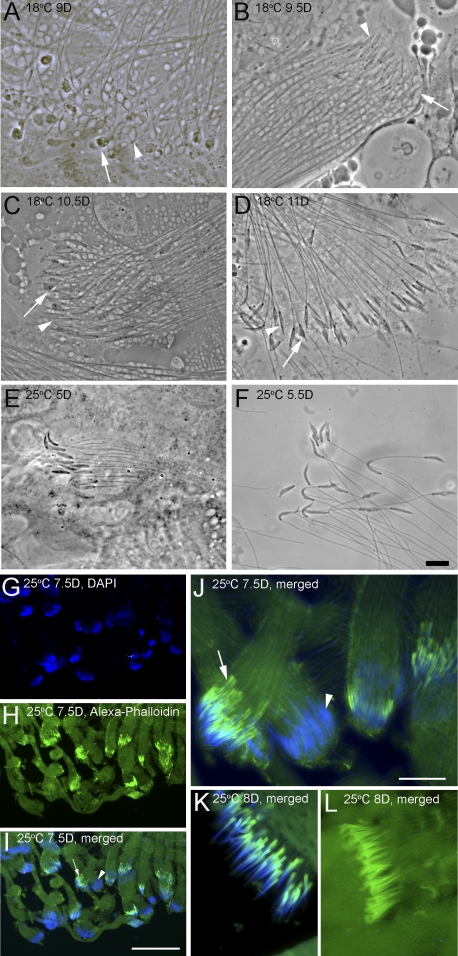
Critical Stage for Temperature Sensitivity Five spermatid bundles of the most advanced stage from each of the five gonads or testes were examined at each critical point of the temperature shift experiment under phase contrast optics (A–F) or epifluorescence (G–L). Spermatid development stages (p to s) were classified according to the standard described in [41]. Scale bar: 10 μm (A–F); 100 μm (G–I); 20 μm (J–L). (A–D) Spermatogenesis at 18 °C before the early middle stage of elongation (stages p or q) can be rescued for *sex-ratio* by shifting to 25 °C (A; also see Figure 6). However, when spermatogenesis reaches late middle stage of elongation (stage r), the rescue is almost impossible (B–D). Thus, stage r is the late boundary of temperature sensitivity. Note the phase dark nucleoplasmic vacuoles in the abnormal nuclei (arrow) as compared to normal nuclear transformation (arrowhead). (E and F) Spermatogenesis at 25 °C up to the middle stage of elongation (stages q and r) can be affected by 18 °C treatment to give full *sex-ratio* (E). However, the temperature sensitivity decreases once the spermatogenesis reaches the fully elongated stage (stage s) (F). (G–L) Individualization complex (IC) is viewed with Alexa Fluor 488 Phalloidin that binds to F-actin (green) while the nuclei are marked by DAPI (blue). By the beginning of individualization (arrow in I and J, where J is a closeup of I; compared to a younger bundle in which the IC has not formed yet, arrowhead), the 18 °C treatment can only cause a slight sex ratio distortion (Figure 6B). On the 8th day of spermatogenesis at 25 °C, there are numerous spermatid bundles that have initiated individualization (K) or that have the IC traversing along the tails (L). The 18 °C treatment has no effect from this time on.

Are the abnormalities observed at the onion stage the primary developmental lesion of *nmy*? In *Drosophila* spermatogenesis, many mutant phenotypes appear to be similar even though the underlying mutations (primary lesions) have dramatic differences in their normal functions [41]. In this case we took advantage of the temperature-sensitivity to detect the primary lesion under the assumption that it is also sensitive to temperature. In temperature-shift experiments, where the chance of mixing early and late sperm is small and the stages of spermatogenesis can be cytologically determined (see Materials and Methods), the critical developmental stages in spermatogenesis can be inferred directly from the sex ratio data (Figures 6 and 7). Up to the early elongation stage, the abnormal nucleoplasmic vacuoles formed at 18 °C can be eliminated by a temperature shift to 25 °C (Figure 7A), whereas a temperature shift is no longer effective after full elongation has been reached (Figure 7B–7D). For the shifts to lower temperature, 18 °C can still cause sex ratio distortion even if the nuclei have reached the middle elongation stage (Figure E), but a switch to 18 °C has little effect once individualization has commenced (Figure 7F–7L). These observations unambiguously agree with the electron microscopy data that the earliest detectable developmental lesions are indeed the abnormal nucleoplasmic vacuoles and that they have a deleterious effect in disrupting nuclear condensation. We do not know what upstream biochemical processes are involved, but chromatin modulation and traffic across the nuclear envelope are among the primary suspects.

## Discussion

We have presented genetic, molecular, and cytological data characterizing the suppression of the Winters *sex-ratio* in *D. simulans.* We have shown that the Winters *sex-ratio* is polymorphic within natural populations of D. simulans, and that it has no relation to D. sechellia as previously thought. The etiology of the aberrant *sex-ratio* is attributed to the failure of nuclear condensation in the Y-bearing sperm. We mapped and cloned an autosomal suppressor that acts to suppress a *sex-ratio* distorter on the X chromosome. The nucleotide sequence and structure of the suppressor strongly suggests a mechanism involving siRNAs. Remarkably, the evolutionary origin of the suppressor is from a transcript of the *sex-ratio* distorter gene itself. Our work supports the theory that intragenomic conflicts are important evolutionary processes that may ultimately underlie several seemingly unrelated major biological phenomena including Mendelian segregation, genome organization and speciation.

### One Species, Three Systems (At Least)

The cases of *sex-ratio* recorded in D. simulans can be recognized as at least three independent systems, as highlighted below.

#### Paris versus Winters.

For the Paris system, two epistatic distorters have been implicated in the *sn–lz* region [22], which is adjacent to but not overlapping the *lz–v* region where the Winters distorter (*Dox*) has been localized [32]. Other evidence also suggests an older evolutionary age for the Winters system, as detailed in [32].

#### Winters versus Durham.

*Tmy* has been mapped to 89A4 and cloned (YT, unpublished data). It is at different location and has a different sequence than that of *Nmy* (87F3). Furthermore, *Tmy* does not suppress *Dox* (crosses 37 versus 39 in Table S1).

#### Paris versus Durham.

Neither does *Tmy* suppress *SR6*, an X chromosome carrying the Paris distorters [32].

These observations suggest that all three *sex-ratio* systems are distinct. We cannot exclude some evolutionary connections among them that genetic tests so far have failed to reveal. Nonetheless, these observations strongly suggest that genetic variations of *sex-ratio* are not rare, at least in D. simulans. Although one search for genetic variation affecting sex ratio in D. melanogaster was negative [42], positive results in the same species have been reported [43,44]. Some X-linked mutations distorting sex ratio can be readily generated through mutagenesis in D. melanogaster [45,46], but for reasons that are unknown, no clear case of *sex-ratio* has ever been found in natural populations of this species.

The recessive mutant (*sxr*) might well be allelic to *nmy*. This gene can be placed 26.6 cM distal to *H* (74.2) and 34.0 cM proximal to *pe* (121.4), i.e., in the vicinity of *Ubx* (90.3) on one genetic map [47]. Thus *sxr* seems to fall in roughly the same region as *nmy* (Figure 1B, also see Table 6 in [17]). The phenotypes of *sxr* also appear to be very similar to *nmy* in several respects, including temperature sensitivity and abnormal morphology in spermiogenesis [17]. At the ultrastructural level, *sxr* spermatogenesis showed pronounced sperm tail degeneration [48], as we report here for *nmy* (Figure 4J).

On the other hand, some phenotypes of *sxr* were described differently from those of *nmy* reported here. One major discrepancy is in the temperature-sensitive stage, which was inferred to be the primary spermatocyte for *sxr* and nuclear transformation for *nmy*. The determination of the *sxr* temperature-sensitive stage could be somewhat discrepant, because the temperature-shift protocol used does not stage spermatogenesis with great precision. In addition, the time course of spermatogenesis inferred for D. simulans was not based on direct cytological examination but was assumed to be the same as that in D. melanogaster [17]. Under TEM, *sxr* males showed abnormalities in the primary spermatocytes and in the axonemal complex before the individualization stage [48], but *nmy* males did not show these defects, even when special attention was given to these possibilities. In any event, if *sxr* and *nmy* are indeed the same gene, the mutation likely has maintained stable frequency in natural populations of California for many decades.

### The Evolution of *sex-ratio* Suppressor

Intragenomic conflicts are struggles within a genome over hereditary transmission [49]. Meiotic drive is one type of intragenomic conflict in that the driving allele or haplotype has more than 50% representation in next generation. For nuclear genes that freely recombine the evolutionary stable strategy is exact Mendelian segregation [8]. Thus for most of the genome, there is strong evolutionary pressure for selecting modifiers that increase the fidelity of Mendelian segregation. This explains why segregation in meiosis is usually Mendelian, and it also underlies Fisher's argument about the sex ratio based on parental expenditure [8]. However, this logic does not apply to cases where free recombination is inhibited. For example, in the two classic cases of autosomal meiotic drive—*Segregation Distortion* (*SD*) in D. melanogaster [50] and the *t- complex* in Mus musculus [51]— the distorter(s) and insensitive responder(s) are locked together within complex inversions. Similarly, the evolution of sex-linked meiotic drive is facilitated by the lack of recombination between the X and the Y chromosomes. It has been reasoned that *sex-ratio* meiotic drive is a more potent evolutionary force than autosomal drive, based on two arguments [11,12]. First, *sex-ratio* drive can evolve more readily. When the sex chromosomes do not undergo recombination along most of their length, which includes most cases of heteromorphic sex chromosomes, many sex-linked genes can potentially mutate to *sex-ratio* distorter. This is usually not true for an autosomal distorter, because the precondition for its invasion is satisfied only in special circumstances, such as in the centromeric region with its reduced recombination or within inversions. Second, *sex-ratio* has a much greater effect on the rest of genome precisely because it affects the sex ratio, and thus, the transmission rates of different genomic compartments. In addition to favoring modifiers that reduce any fitness cost, *sex-ratio* favors modifiers that render the sex ratio more equal, as mandated by Fisher's principle [52]. The evolutionary cycle of distorter and suppressor could go on indefinitely as long as new *sex-ratio* mutations unaffected by existing suppressors can occur.

When a *sex-ratio* mutation invades a population, its fate can be fixation, stable polymorphism, or extinction, depending on the configuration of fitness components (particularly the fitness of *sex-ratio* males) [53]. Polymorphisms of distorters and suppressors have been reported in several *Drosophila* species, including D. simulans [25], D. quinaria [54,55], D. obscura [56], D. paramelanica [57], and D. mediopunctata [58–60]. Fixation of a suppressor is likely the case for the cryptic *sex-ratio* suppressor *Tmy* in D. simulans [15]. However, no *sex-ratio* suppressors have been found in D. pseudoobscura, and the cause may be related to high fitness cost of *sex-ratio* males [61,62]. It is remarkable in itself that modifiers that increase the fitness of *sex-ratio* males seem not to have been selected for the past 0.7–1.3 My [63]. The answer may lie in the molecular mechanism of this *sex-ratio* distorter that is still unknown. Molecular characterization of the Winters *sex-ratio* system makes possible future studies on the molecular population genetics and ecological dynamics of suppressors in natural populations.

### The Molecular Mechanism of *sex-ratio* and Its Evolutionary Impact

We have shown here that an RNAi mechanism is likely involved in the Winters *sex-ratio* suppression. Remarkably, the suppressor *Nmy* was generated through the use of the sequence information of the *Dox* distorter itself by means of retrotransposition. An interesting observation is that the abnormal transformation of the Y-bearing sperm in *Dox*;*nmy* male is exacerbated at lower temperature. If a lack of small RNAs encoded by the *nmy* allele is indeed the cause for derepressing the expression of *Dox*, then the molecules encoded by *Dox* (RNA or protein) may be more harmful to the *Y*-bearing sperm at lower temperature. Further in-depth molecular analysis of this hypothesis is required. This *Dox*-*Nmy* system is reminiscent of a possible case of cryptic *sex-ratio* system *Ste/Su*(*Ste*) in D. melanogaster [64,65]. The distorter *Ste* and the suppressor *Su*(*Ste*) share common sequences, and an RNAi mechanism has been convincingly shown to be responsible for the suppression [66,67].

The cytological defects in *nmy* are also reminiscent of abnormal nuclear condensation in the classical meiotic drive system *SD* in D. melanogaster [50]. The gene *Sd* targets the *Responder* (*Rsp*) locus and causes degeneration of *Rsp*-bearing sperm [68]. The *Sd*-bearing sperm is thus transmitted to more than 95% of the progeny from *Sd*/*Rsp* males. *Sd* is a truncated duplication of RanGAP and is still enzymatically active [69,70]. It may cause problems in establishing a normal RanGTP–RanGDP gradient across the nuclear envelope [71], which is a critical condition for many cytological functions including transport of small RNAs [72]. The *Rsp* locus consists of a cluster of satellite repeats whose sensitivity is proportional to copy number [73]. One might speculate that an interruption of heterochromatin condensation in and around the *Rsp* cluster directly causes the degeneration of *Rsp*-bearing sperm, because normal heterochromatin regulation requires small RNAs homologous to the heterochromatic region [74], whereas small RNAs originating and/or targeting the *Rsp* cluster may be misregulated in *Sd/Rsp* males. Indeed, small RNAs originated from the *Rsp* locus have been recorded [75].

Segregation distortion in males may happen at either meiotic or post-meiotic stages. For example, the Y chromosome can be fragmented or lost during meiosis II [29,76], or the Y-carrying spermatids may not mature [77,78]. Post-meiotic failure in spermiogenesis can result from aberrations in meiosis. For example, failure of X-Y pairing during prophase of meiosis I has been shown to cause spermiogenic failure in D. melanogaster [77,79]. For the Winters *sex-ratio,* it is unclear whether there is any pairing problem between the sex chromosomes, and whether chromosomal behavior during meiosis is normal. Through light and electron microscopy, we have demonstrated that the primary lesion in *nmy* mutants is a defect in nuclear condensation during spermiogenesis. In any case, segregation distortion in males usually results from a failure to produce a class of sperm, or a failure of a class of sperm to function, and there is a concomitant reduction in fertility.

Whatever the actual molecular mechanisms or developmental stages involved, it is logical to make an evolutionary link between *sex-ratio* meiotic drive and speciation. A meiotic drive distorter first exploits the meiotic and post-meiotic mechanisms for the biased transmission of itself, then the genome regains balanced transmission through the evolution of suppressors that correct the meiotic or post-meiotic aberrations. During each episode of distortion and suppression, male fertility might be compromised and recovered. Because of these dynamics, the meiotic/post-meiotic genes have an accelerated rate of evolution, thus promoting the reproductive isolation among isolated populations or incipient species that do not share common distorters and suppressors [13]. Under this meiotic drive scenario, it is easy to explain two ubiquitously observed genetic patterns for postzygotic reproductive isolation: a much faster accumulation of hybrid male sterility among *Drosophila* species, and a “large X” effect for the distribution of the hybrid male sterility genes [13]. Two *Drosophila* cases where one gene expresses both hybrid male sterility and *sex-ratio* lends direct support to a role for meiotic drive in speciation [14,15]. A testable prediction is that genes that are responsible for the initial postzygotic reproductive isolation between species most likely function in meiotic and post-meiotic processes such as chromosome segregation and chromatin condensation.

## Materials and Methods

### Fly stocks.

Five *NSR* (normal sex ratio) lines (IG88, IG113, IG118, IG132, and IG143) and six *SSR* (skewed sex ratio) lines (IG12, IG33, IG54, IG151, IG73, and Q15.3) were used from a previous D. simulans × D. schellia hybridization in which the parental D. simulans stock, sim2, had been collected in Winters, California [20]. The D. mauritiana × D. simulans introgression lines have been described before [13,31]. The following lines were used in this study: homozygous introgression line *P*18.16 and *P*38.7 and heterozygous line *P*40.6. The heterozygous 2-*P* lines were made by recombining two *P*[*w* ^+^] inserts in *cis* from two introgression lines: *2P-10* (*P*40.8 × *P*38.4), *2P-20* (*P*37.1 × *P*35.2), *2P-19* (*P*37.3 × *P*38.5), *2P-17* (*P*35.2 × *P*46.17), and *2P-15* (*P*38.1 × *P*46.17). The expression of the *w* ^+^ allele affecting eye color in *P*[*w* ^+^] is sensitive to its position and copy number. Essentially, these *P*[*w* ^+^] inserts provide semi-dominant markers along the third chromosome in D. simulans.

A D. simulans stock with the multiply marked third chromosome *jv st e pe* has been described before [80]. These mutations and their genetic positions are *javelin* 3–19.2, *scarlet* 3–49.5, *ebony* 3–63.0, and *peach* 3–104.9 [81]. They are allelic to D. melanogaster mutations *javelin* 3–19.2, 65A5-E1 [82], *scarlet* 3–44.0, 73A3 [82], *ebony* 3–70.7, 93C7-D1 [47], and *pink* 3–48.0, 85A6 [82], respectively. Note that because there is a large inversion 84F-94F on 3R in D. simulans as compared with D. melanogaster, the map positions of *e pe* in D. simulans are reversed on the map of D. melanogaster. Other D. simulans stocks *simB* (*w; nt; III*) and *w; e* have been described before [31]; and *C(1)RM y w*/*lz^S^* was kindly provided by J. Coyne.

### Fly work.

All flies were reared on cornmeal-molasses-agar medium sprinkled with yeast grains at room temperature (22 ± 1 °C) if not otherwise indicated. The *sex-ratio* phenotype of a male was scored by mating the male with three tester virgin females, usually of the stock *w; e*, for 7 d before clearing all adults. The progeny were sexed and counted three times until the 19th day. The sex ratio (*k*) was calculated as the proportion of females.

### Molecular biology.

The use of ASO markers was described previously [31]. Some key techniques and the reagents/kits used are as follows: long PCR (Takara LA Taq); PCR product cloning (Topo TA and Topo XL PCR cloning kits, Invitrogen); sequencing of large DNA fragment (EZ-Tn5 Insertion Kit, Epicentre); phage genomic library of *D. simulans simB* (Lambda ZAP II vector predigested with *Eco*R I, stratagene); RNA isolation (TRIZOL Reagent, Invitrogen); RT-PCR (reverse transcription polymerase chain reaction) (3′ and 5′- RACE kits and SuperScirpt II Reverse Transcriptase, Invitrogen).

### Light microscopy.

Testes or gonads from crawling larvae or young adult males were dissected into saline (0.7% NaCl). Spermatids or mature sperm were released from gonads/testes with a fine tungsten needle. Live specimens were observed directly under phase contrast. For a reliable count of abnormal spermatids, the specimens were fixed for 20 s to 1 min on a microscope slide with 10 μl 2% glutaraldehyde in phosphate-buffered saline (PBS) (2.7 mM KCl, 137 mM NaCl, 8.0 mM KH_2_PO_4_); and rinsed with PBS for 5 min before staining with DAPI (100 ng/ml in PBST–0.1% Triton X-100 in PBS) for 5 min. The specimens were spread with vigorous tapping on the cover slip before being observed under epifluorescence. To visualize the IC [83], an additional step was added in the above protocol: fixed specimens were rinsed for 5 min in PBSTB (PBS with 0.1% Triton X-100 and 1% BSA) and stained 10 min with Alexa Fluor 488 Phalloidin (10 μ/ml; Invitrogen). The specimens were rinsed again for 3 min with PBSTB before DAPI staining. For longer storage, specimens were mounted in SlowFade Gold (Invitrogen). Images were taken on an Axioskop2 or Leica DMRB with digital camera, and processed with Adobe Photoshop.

### TEM.

In a drop of chilled 0.067 M pH 7.4 phosphate buffer, testes and accessory glands were dissected from young males (1–3 d old) with a fine tungsten needle and were transferred immediately to 2% glutaraldehyde in 0.067 M phosphate buffer on ice. The specimens were fixed for 2 h at 4 °C in 1% paraformaldehyde and 2% glutaraldehyde in 0.067 M phosphate buffer, followed by a post-fixation of 1 h in 2% OsO4 at 4 °C. The specimens were treated with 1% uranyl acetate at room temperature for 1 h before processing through an ethanol series to dehydrate. The specimens were trimmed after ethanol dehydration so that only one of each pair of testes was used in embedding. The Epon resin was made by mixing Taab Epon (42.4 g), DDSA (19.8 g), and NMA (18.0 g) for 30 min before adding 1.9 g DMP-30. Before final embedding, each testis was cut into 4–5 segments and aligned on the bottom of the mold in a straight line with the apical tip facing out. Sections were cut on a Reichert ultracut-S microtome, followed by staining with uranyl acetate and lead citrate. The grids were observed with a Tecnai G^2^ Spirit BioTWIN electron microscope.

### Temperature shift experiments.

Vials with *SSR*12-2-7 (*nmy*) eggs collected at 25 °C were cultured at 18 °C or 25 °C and shifted to the other temperature at time points of 0.5 or 2 d apart (0.5 d for the more critical stages) until adult flies emerged. The first few males after eclosion from each vial were singly mated to ten *w; e* 2-d-old virgins, which were adequate to exhaust the sperm of a single male (unpublished data). Each male was aspirated to new vials containing 10 virgin females every day for 3–5 d. Offspring from each vial were counted, but only those from the first vials were used in data analysis once their total reached over 50. On average, 148 and 108 offspring per male were used for calculating the sex ratio for the 18 °C to 25 °C shift and the 25 °C to 18 °C shift, respectively. In this way the first batch of sperm developing from the first few sperm bundles were exhausted and assayed. Critical intervals that were sensitive to temperature were detected from the change in sex ratio. Furthermore, the spermatogenetic stages of the first most mature bundles at these sensitive intervals were determined cytologically, either through phase contrast optics as described in D. melanogaster [41], or using Alexa Fluor 488 Phalloidin for viewing the individualization complex [83].

## Supporting Information

Figure S1 The Gene Responsible for *sex-ratio* Is Recessive and Maps to the Third Chromosome *SSR12* expresses sex ratio distortion while *Cy; Antp* does not. However, this experiment does not rule out the possibility that the expression of *sex-ratio* is conditioned on the presence of the X and/or the second chromosomes from the *SSR* lines.
(245 KB PDF)

Figure S2 Extraction of the Third Chromosome into the *simB w; nt; III* Background (A) Five third chromosomes were extracted for each of the three *SSR* lines (IG12, IG54, and IG151) and three *NSR* lines (IG88, IG113, and IG118). Two different *P*-element inserts (*P*18 and *P*40), which are distinguishable by eye color, were used as dominant markers in this cross scheme. The introgressed D. mauritiana material is shown in gray [31] but was irrelevant to our purpose here. There might be recombination in the females, thus flies in *G4* are not necessarily all homozygous for the *SSR* or *NSR* alleles. However, the majority of the vials from *G4* showed *sex-ratio* for the *SSR* lines, but only a few vials showed *sex-ratio* for the *NSR* lines (B). The overall average clearly indicates that the responsible gene is located on the third chromosome. Several lines from each of the three *SSR* lines were stably expressing *sex-ratio* up to *G10*, but only one from each of the *SSR* lines (*SSR12-2-7*, *SSR54-2-3*, and *SSR151-2-10*) was kept for further analysis.
(288 KB PDF)

Figure S3 Extraction of the Second Chromosome into the *simB w; nt; III* Background (A) Five second chromosomes were extracted for each of the three *SSR* lines (IG12, IG54, and IG151) and three *NSR* lines (IG88, IG113, and IG118). In *G5*, 1/3 of *nt* ^+^ males should be +/+. The proportion is higher from vials where no *nt* flies were found in their progeny. If the genes on the second chromosome are responsible for *sex-ratio*, there should be *sex-ratio* in the *nt* ^+^ class as compared to *nt* class (B). There is no indication of *sex-ratio* in any of these genotypes, suggesting that no genes on the second chromosome are involved.
(326 KB PDF)

Figure S4 Extraction of the X Chromosome into the *CM(1) y w*/*lz* ^s^ Background (A) Five sublines were set up for *SSR12* and *NSR88* by mating single males to ten *CM(1) y w* females. Note that sex ratio in this generation was calculated as the proportion of males. In the F_1_ and subsequent backcrosses, three males were crossed to ten *CM(1) y w* females. In the F_1_, BC_1_, BC_5_, and BC_10_ generations, three males from each subline were singly mated to three *w; e* virgins, and the sex ratios of their progeny were scored (B). The X chromosome clearly does not differ between *SSR12* and *NSR88* with respect to sex ratio.
(271 KB PDF)

Figure S5 Mapping the Gene on the Third Chromosome (A) In all crosses, females are at the left-hand side. Crosses *G1–G3* were set up en masse (10–12 pairs of virgin males and females). Cross *G4* was set up by singly mating to three *sim2* virgin females. In *G3*, males and females of all the 16 possible genotypes of the four markers were mated. In *G4*, some males are heterozygous for some markers, whereas their phenotype is not distinguishable from wild-type homozygotes. Because the target gene (*) is recessive, homozygote of closer “+” markers will have a higher chance of expressing *sex-ratio*. A total of 1342 males were tested. Region surrounding *pe* is thereby implicated with gene affecting *sex-ratio* (B).
(284 KB PDF)

Figure S6 The 2-*P* Mapping Scheme (A) The mapping target is determined by its association with either of the two *P*-elements, whose eye color phenotypes are easy to distinguish from each other and from flies with both *P*-elements. The *sex-ratio* phenotype of each recombinant was tested by complementation with *SSR12-2-7* that is homozygous for the mapping target, a recessive loss-of-function suppressor (*). The two *P* inserts and the region of introgressed D. mauritiana of each 2-*P* lines are shown in Figure 1B. Recombinants with either (like *P*40 or *P*38) or both (like *2P*10) *P* inserts host the loss-of-function allele (*) if *k* > 0.5 (B). Clearly, the target (*) is localized in between *P*40 and *P*38, an interval of ∼2,700 kb, as estimated by determining the exact *P-*element insertion sites through inverse PCR (Araripe L, Eckstrand N, Hartl DL, Tao Y, unpublished data).
(286 KB PDF)

Table S1 Sex Ratio Tested for Various Genotypes (97 KB DOC)

Table S2 New ASO Markers Developed in this Study (116 KB DOC)

Table S3 ASO Probes in the Last 7-kb Region of Fine Mapping (56 KB DOC)

Table S4 Viability from Egg to Adult (58 KB DOC)

Text S1 A Recessive *sex-ratio* Suppressor on the Third Chromosome (43 KB DOC)

Text S2 Relevant Primers Used (20 KB DOC)

Text S3 Transcripts of *Nmy*[*sim2041*] and *nmy*[*sim1427*] (30 KB DOC)

### Accession Numbers

All sequences have been deposited in the GenBank (http://www.ncbi.nlm.nih.gov/Genbank/index.html) database and have been assigned the accession numbers EF565211–EF565217.

## Abbreviations

ASO: allele-specific oligonucleotide
DAPI: 4',6-diamidino-2-phenylindole
IC: individualization complex
IR: inverted repeats
RACE: rapid amplification of cDNA ends
*SD,*: *Segregation Distortion;*
siRNA: small interfering RNA
TEM: transmission electron microscopy
