# Supplementary material for: A sex-ratio Meiotic Drive System in Drosophila simulans. I: An Autosomal Suppressor
Source: PLoS Biol. 2007 Nov 6;5(11):e292. doi: 10.1371/journal.pbio.0050292 (PMC2062475; doi:10.1371/journal.pbio.0050292)
Supplement: Supplementary file 1 [file pbio.0050292.sg001.pdf]

Figure S1.

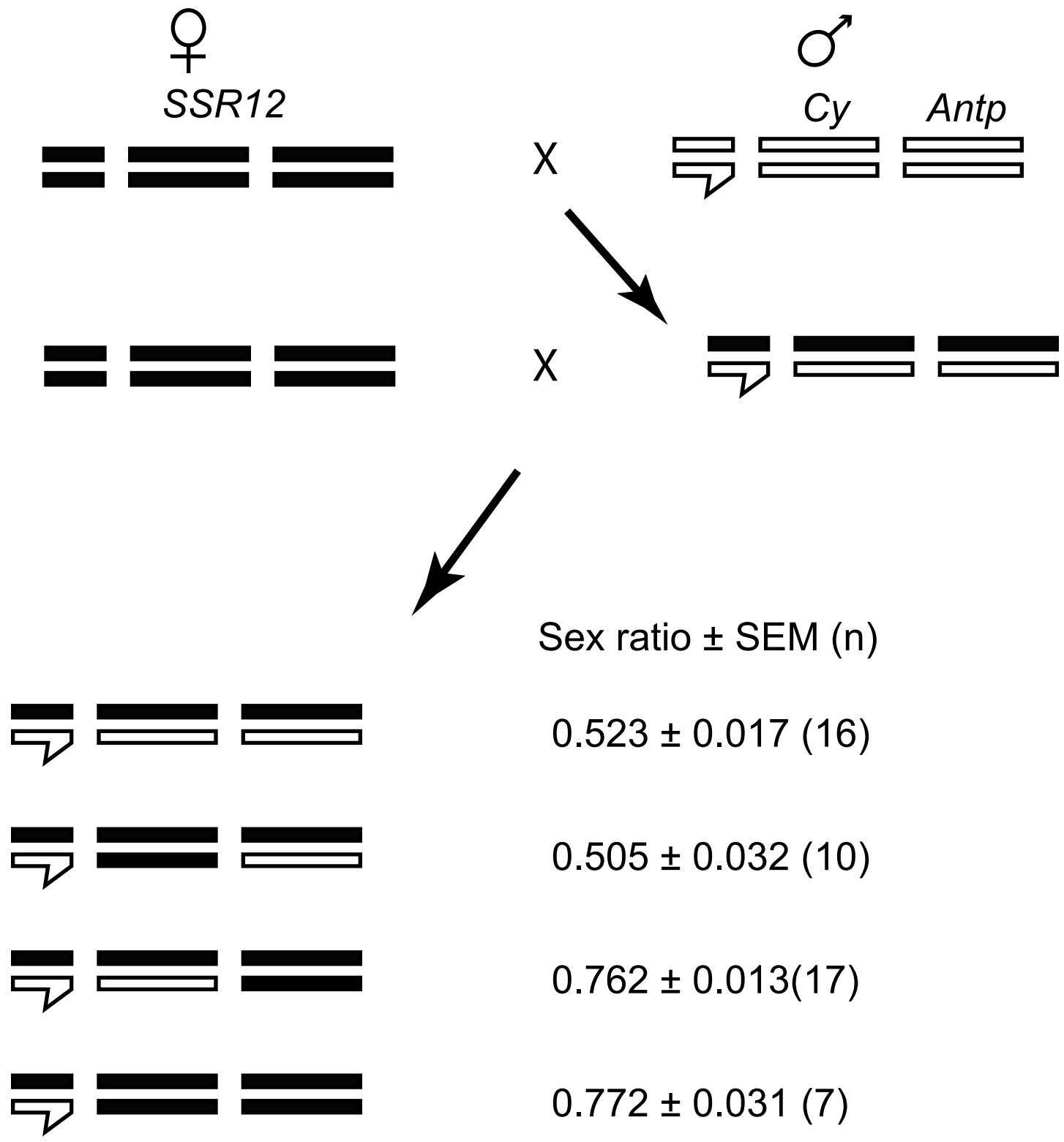
♀
SSR12
X
♂
Cy
Antp
X
Sex ratio ± SEM (n)
0.523 ± 0.017 (16)
0.505 ± 0.032 (10)
0.762 ± 0.013(17)
0.772 ± 0.031 (7)
