# Supplementary material for: A sex-ratio Meiotic Drive System in Drosophila simulans. I: An Autosomal Suppressor
Source: PLoS Biol. 2007 Nov 6;5(11):e292. doi: 10.1371/journal.pbio.0050292 (PMC2062475; doi:10.1371/journal.pbio.0050292)
Supplement: Supplementary file 2 [file pbio.0050292.sg002.pdf]

Figure S2.

A

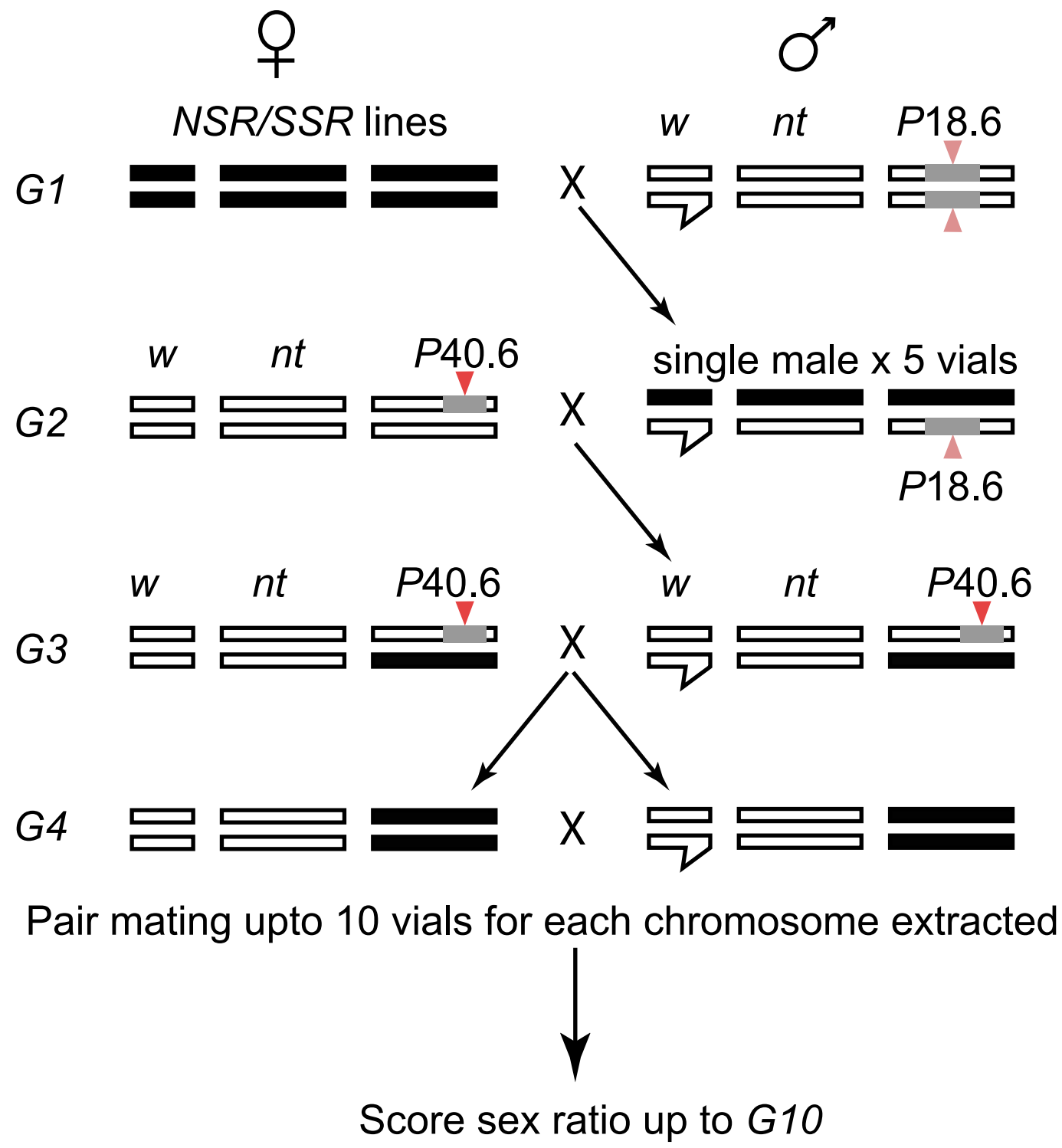

B

Sex ratio scored at *G5*

Lines	# Chromosome extracted	#Vials	#Vials with *k* > 0.5 (*P* = 0.05)	Overall		
				Male	Female	*k*
SSR12	5	46	32	311	1518	0.830
SSR54	4	29	19	49	442	0.900
SSR151	5	29	24	52	700	0.931
NSR88	4	35	2	709	740	0.511
NSR113	5	39	1	470	480	0.505
NSR118	5	41	6	754	904	0.545

The *k* values between *SSR* and *NSR* lines are significantly different (*P* << 0.001, one way ANOVA)
