# Supplementary material for: A sex-ratio Meiotic Drive System in Drosophila simulans. I: An Autosomal Suppressor
Source: PLoS Biol. 2007 Nov 6;5(11):e292. doi: 10.1371/journal.pbio.0050292 (PMC2062475; doi:10.1371/journal.pbio.0050292)
Supplement: Supplementary file 3 [file pbio.0050292.sg003.pdf]

Figure S3.

A

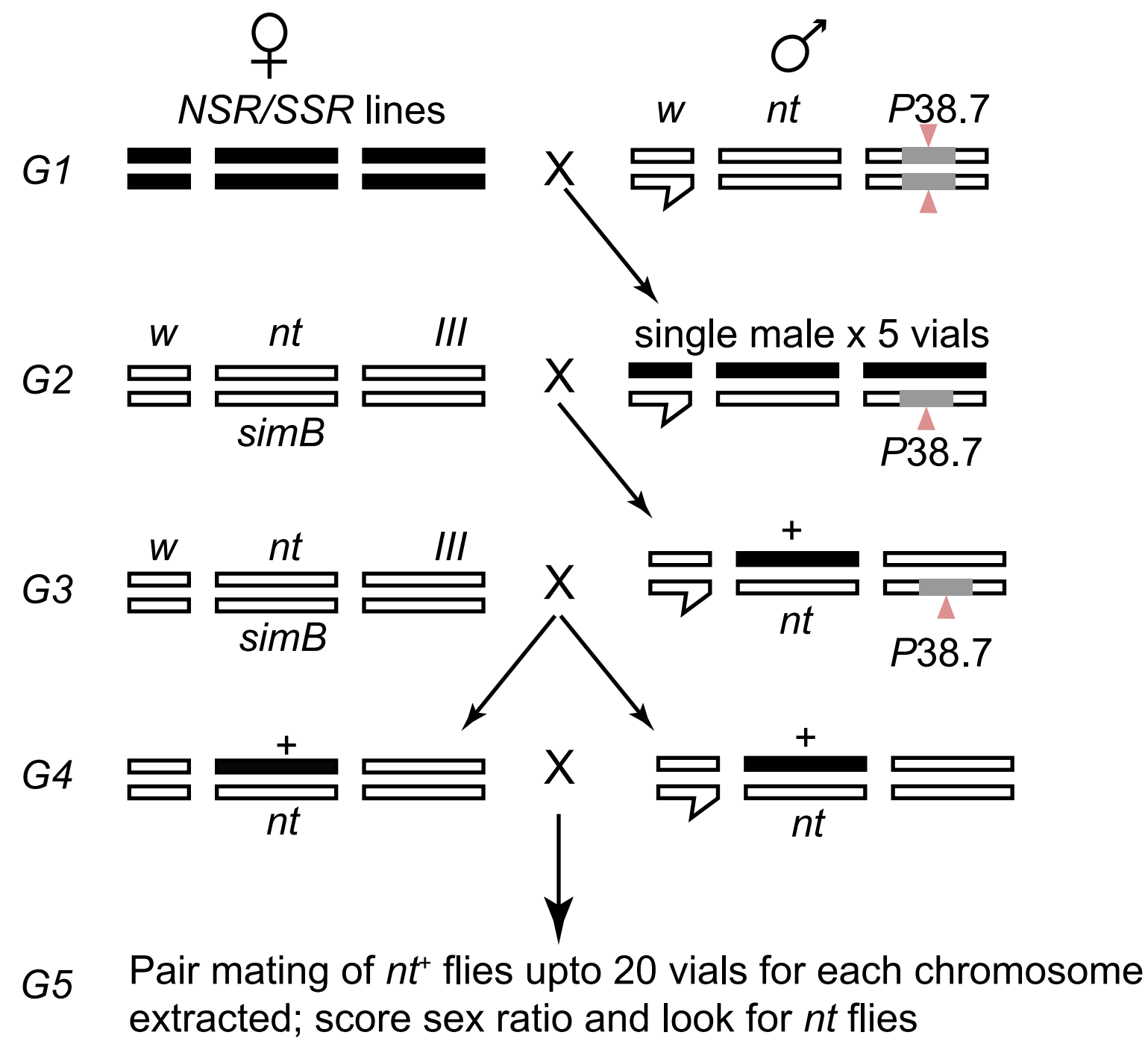

B

Sex ratio scored at *G6*

Lines	# Chromosome extracted	*nt*+ or *nt* (*)	# Vials	# Vials with *k* > 0.5 (*P* = 0.05)	Overall		
					Male	Female	*k*
SSR12	5	*nt*	33	3	455	501	0.524
		nt+	35	5	414	562	0.576
SSR54	5	*nt*	27	0	439	444	0.503
		nt+	39	1	538	509	0.486
SSR151	5	*nt*	38	0	645	604	0.484
		nt+	50	0	817	760	0.482
NSR88	5	*nt*	28	3	396	441	0.527
		nt+	37	3	357	419	0.540
NSR113	5	*nt*	41	1	605	601	0.498
		nt+	26	3	449	485	0.519
NSR118	4	*nt*	30	4	447	473	0.514
		nt+	18	0	137	169	0.552

The *k* values between *SSR* and *NSR* lines are not significantly different (*P* = 0.313, one way ANOVA).

* *nt* indicates the vials where the *nt* flies were found at *G6*. Since the sample size was usually small, *nt* does not necessarily include all +/*nt* × +/*nt* pair mating at G5. However, nt^+ should have 1/3 of vials with homozygous male (+/+) in the pair mating.
