# Supplementary material for: A sex-ratio Meiotic Drive System in Drosophila simulans. I: An Autosomal Suppressor
Source: PLoS Biol. 2007 Nov 6;5(11):e292. doi: 10.1371/journal.pbio.0050292 (PMC2062475; doi:10.1371/journal.pbio.0050292)
Supplement: Supplementary file 4 [file pbio.0050292.sg004.pdf]

Figure S4.

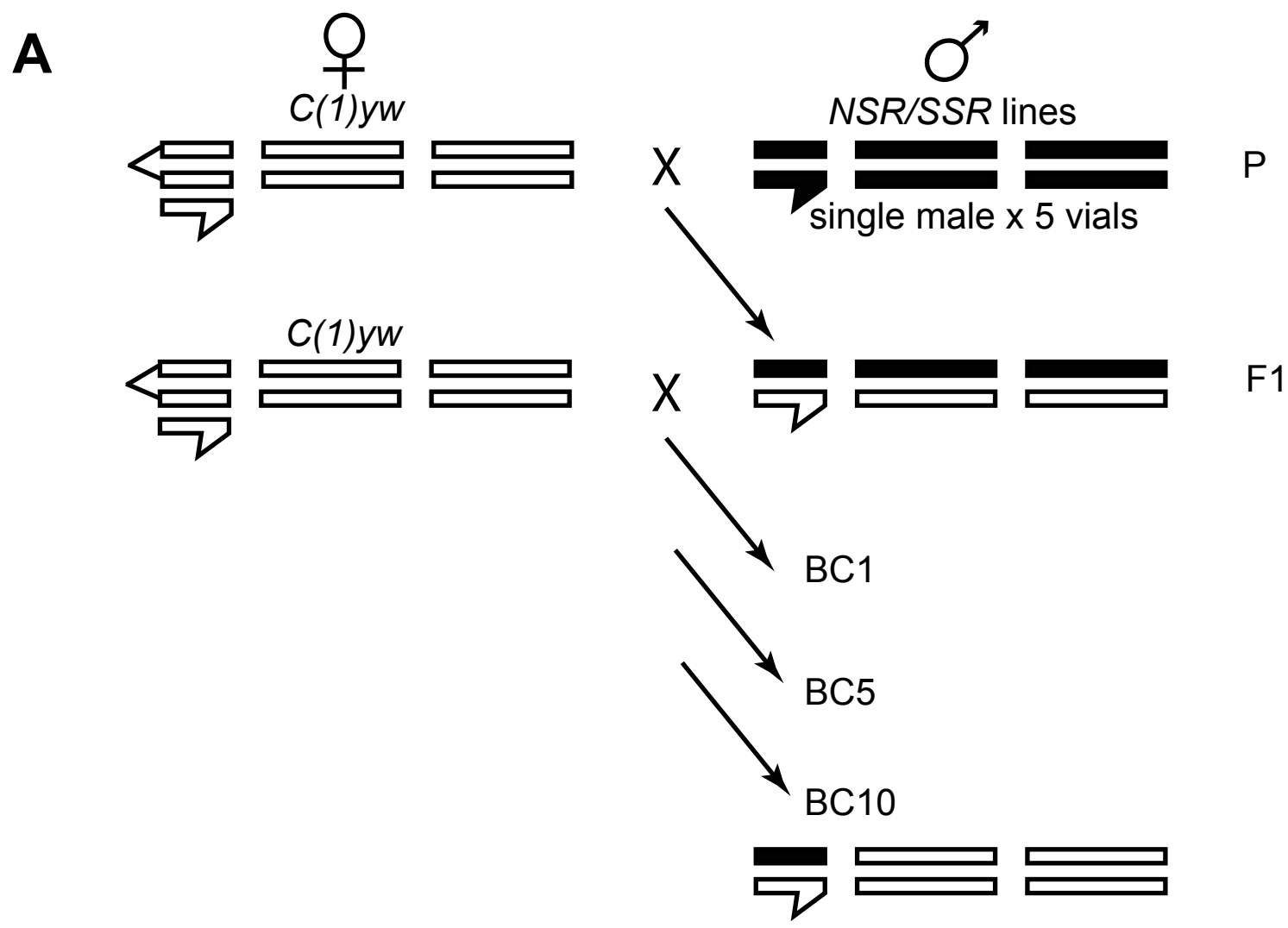

B Sex ratio scored along the backcrossing of *SSR*12 and *NSR*88

Generations	k (mean ± SEM)[b]	
	SSR12	*NSR88*
P[a]	0.795 ± 0.024	0.526 ± 0.028
F_1	0.501 ± 0.010	0.498 ± 0.016
BC_1	0.486 ± 0.016	0.479 ± 0.011
BC_5	0.466 ± 0.016	0.502 ± 0.031
BC_{10}	0.492 ± 0.007	0.483 ± 0.013

[a] In this generation k was calculated as proportion of male.

[b] No significant differences between k's from all generations (ANOVA, $P > 0.05$) except in the P generation (ANOVA, $P = 0.00008$).
