# Supplementary material for: A sex-ratio Meiotic Drive System in Drosophila simulans. I: An Autosomal Suppressor
Source: PLoS Biol. 2007 Nov 6;5(11):e292. doi: 10.1371/journal.pbio.0050292 (PMC2062475; doi:10.1371/journal.pbio.0050292)
Supplement: Supplementary file 5 [file pbio.0050292.sg005.pdf]

Figure S5.

A

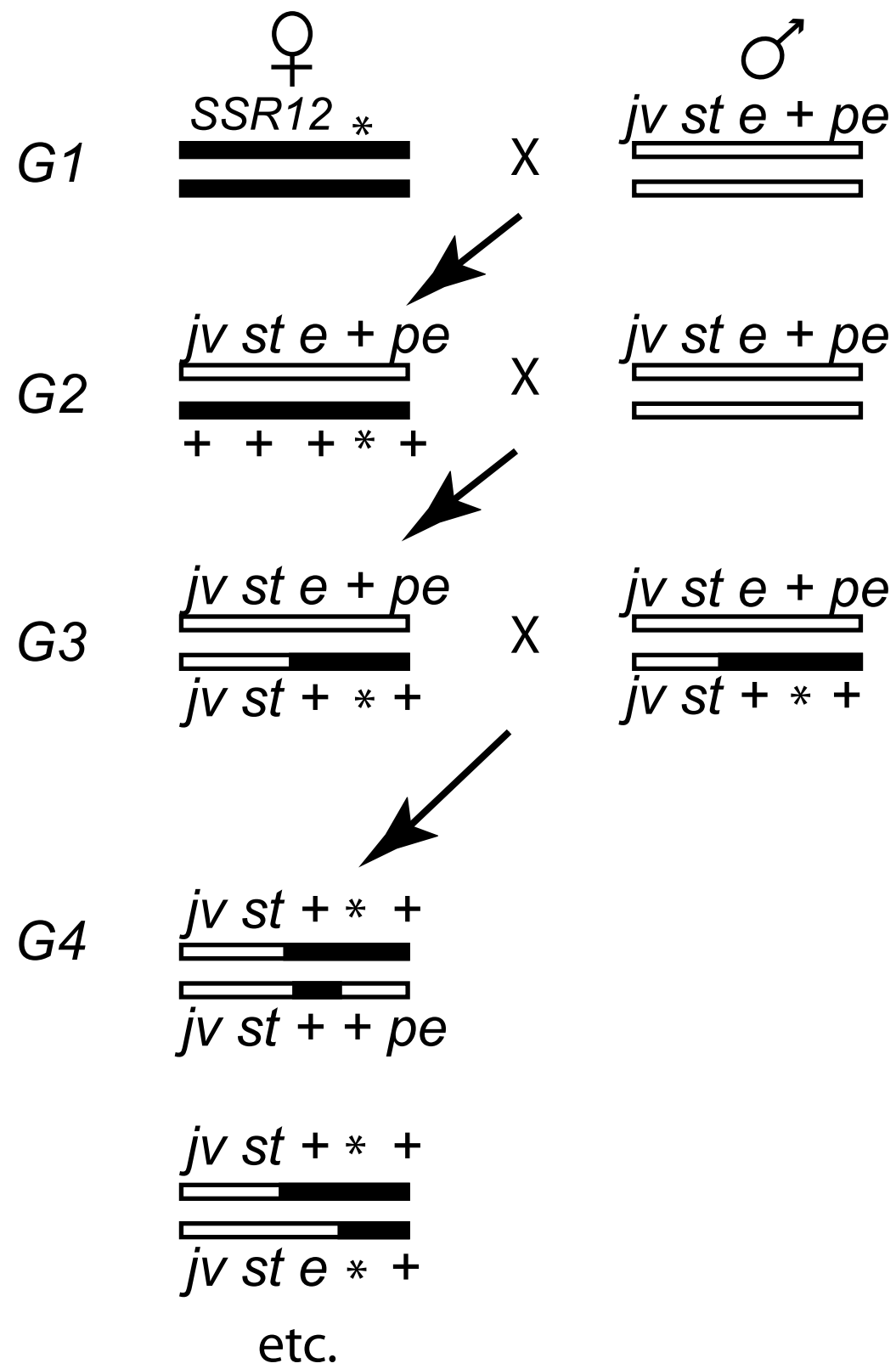

B

Association of Markers with *sex-ratio* Gene (or Genes) on the third Chromosome through ANOVA

Markers	Df	Mean Square	*F*-value	*P*-value
jv	1	137.895	3.633	0.0569
st	1	79.228	2.087	0.1487
e	1	218.065	5.745	0.0167
pe	1	883.298	23.273	<0.0001
jv × *st*	1	3.04	0.08	0.7772
jv × *e*	1	110.67	2.916	0.0879
jv × *pe*	1	65.382	1.723	0.1896
st × *e*	1	9.406	0.248	0.6187
st × *pe*	1	0.25	0.007	0.9353
e × *pe*	1	62.823	1.655	0.1985
jv × *st* × *e*	1	15.962	0.421	0.5168
jv × *st* × *pe*	1	57.306	1.51	0.2194
jv × *e* × *pe*	1	16.82	0.443	0.5057
st × *e* × *pe*	1	12.727	0.336	0.5622
jv × *st* × *e* × *pe*	1	70.538	1.859	0.173
Residual	1326	37.954		
