# Supplementary material for: A sex-ratio Meiotic Drive System in Drosophila simulans. I: An Autosomal Suppressor
Source: PLoS Biol. 2007 Nov 6;5(11):e292. doi: 10.1371/journal.pbio.0050292 (PMC2062475; doi:10.1371/journal.pbio.0050292)
Supplement: Supplementary file 6 [file pbio.0050292.sg006.pdf]

Figure S6.

A

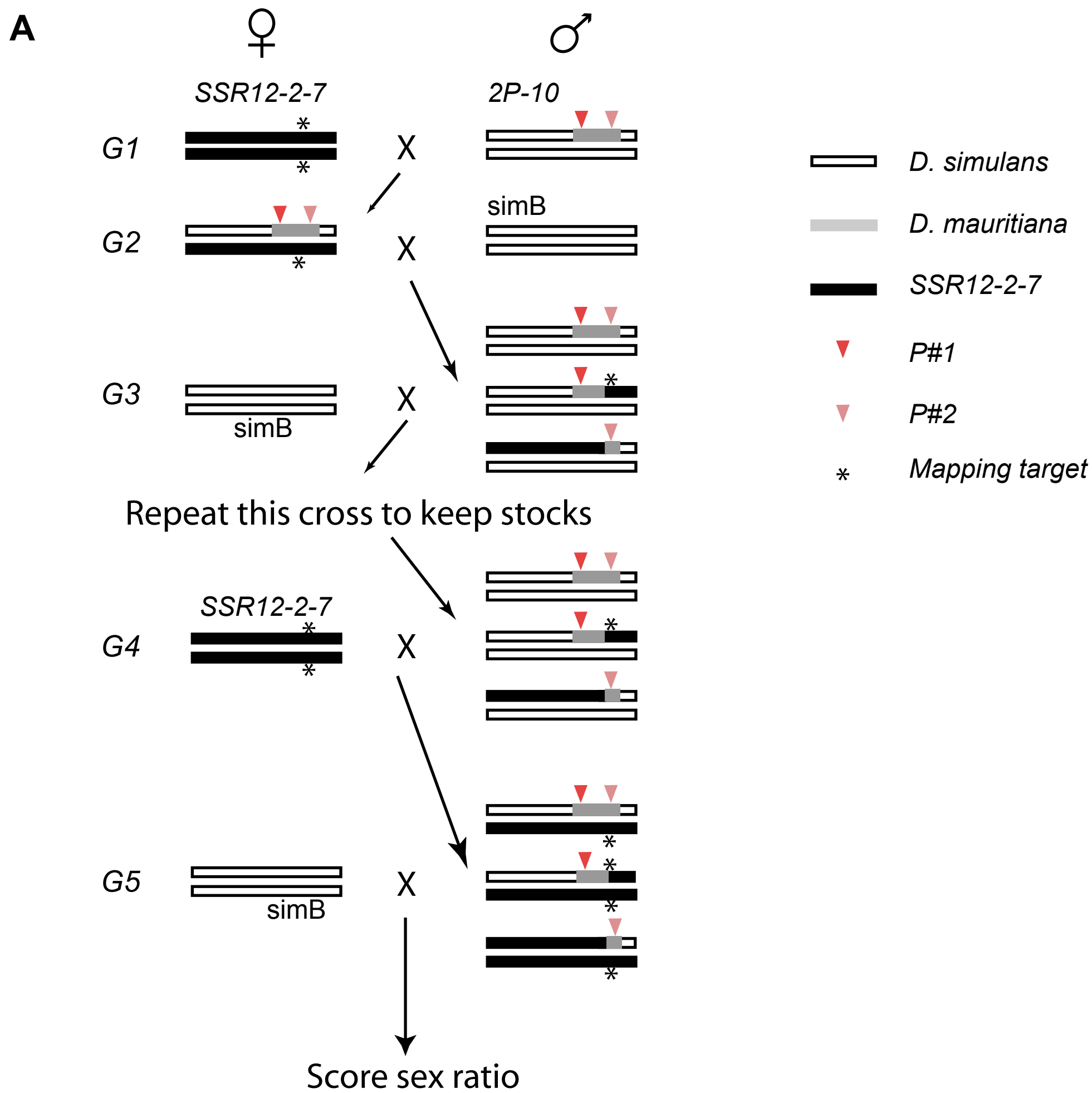

B

2-*P* lines	Recombinants	n	#Recombinants[a] with *k* > 0.5
2P-10	*2P*10	70	0
	*P*40	56	51
	*P*38	73	4
2P-20	*P*37	42	0
	*P*35	45	45
2P-19	*P*38	43	0
	*P*37	40	40
2P-17	*2P*17	68	4
	P46	73	73
	P35	55	1
2P-15	*2P*15	84	0
	*P*38	87	0
	*P*46	88	88

[a]At the level of *P'* = 0.05/n where n is the number of recombinants.
