# Supplementary material for: A sex-ratio Meiotic Drive System in Drosophila simulans. I: An Autosomal Suppressor
Source: PLoS Biol. 2007 Nov 6;5(11):e292. doi: 10.1371/journal.pbio.0050292 (PMC2062475; doi:10.1371/journal.pbio.0050292)
Supplement: Supplementary file 7 [file pbio.0050292.sd001.doc]

**Text S1.** A Recessive *sex-ratio* Suppressor on the third Chromosome

The initial genetic analysis of the Winters *sex-ratio* started with four skewed sex ratio (*SSR*) lines (IG12, IG33, IG54, and IG151) and three normal sex ratio (*NSR*) lines (IG88, IG113, IG118) from a previous study (Dermitzakis *et al*. 2000. *Genetics* 154: 687-694). In one cross, we showed that at least one recessive gene on the third chromosome was responsible for revealing the *sex-ratio* trait (Figure S1), with the caveat that modifiers may also present on the *X* and/or the second chromosome. We excluded this possibility by showing that only the third chromosome is involved in revealing the *sex-ratio* trait (Figure S2), while the *X* and the second are irrelevant (Figures S3 and S4). Since the original *SSR* lines are heterogeneous, we extracted the third chromosome to an isogenic background of simB (*w; nt ; III*) (Figure S2A). The three extraction lines, *SSR12-2-7*, *SSR54-2-3* and *SSR151-2-10*, have been used for further analyses. Complementation tests confirmed that these three lines have the same gene controlling *sex-ratio*, and that this gene is recessive to the wild type allele in the stock simB (*w; nt ; III*) as well as to the *D. mauritiana* allele in the *D. mauritiana* × *D. simulans* introgression lines (Table S1). This gene does complement *too much yin* (*tmy*), a *D. mauritiana* allele introgressed into *D. simulans* background associated with male infertility and *sex-ratio* (Tao *et al*. 2001. *Proc. Natl. Acad. Sci*. 98: 13183-13188) (Table S1). We further used visible markers *jv st e pe* to localize this gene to near *pe*, which is allelic to *pink* (3-48, 85A6) in *D. melanogaster* (Figure S5).
