# Supplementary material for: A sex-ratio Meiotic Drive System in Drosophila simulans. I: An Autosomal Suppressor
Source: PLoS Biol. 2007 Nov 6;5(11):e292. doi: 10.1371/journal.pbio.0050292 (PMC2062475; doi:10.1371/journal.pbio.0050292)
Supplement: Supplementary file 8 [file pbio.0050292.sd002.doc]

**Text S2.** Relevant Primers Used

For *D. simulans* and *D. mauritiana*,

K44.F30a	ACGCAGCATTGCAAATATTTTTATGACACG

K50.R30a	GATGTAAAAACGGATTTGCTGTCATTGGAG

For *D. sechellia*, K44.F30b, which is 269bp distal, is used to replace K44.F30a.

K44.F30b	gaacgagtatcaggtatgttaaccggcgat
