# Supplementary material for: A sex-ratio Meiotic Drive System in Drosophila simulans. I: An Autosomal Suppressor
Source: PLoS Biol. 2007 Nov 6;5(11):e292. doi: 10.1371/journal.pbio.0050292 (PMC2062475; doi:10.1371/journal.pbio.0050292)
Supplement: Supplementary file 9 [file pbio.0050292.sd003.doc]

**Text S3.** Transcripts of *Nmy*[*sim2041*] and *nmy*[*sim1427*]

We have obtained various RT-PCR products from *Nmy*[*sim2041*] and *nmy*[*sim1427*] but some were obviously spurious RT products for the following two reasons: First, the splicing donor and acceptor sites are not canonical (GT … AG), and these sites are all outside of the inverted repeat region. Second, an unusually high rate of point mutations (A→G) was observed from these transcripts in the IR31 region, a telltale of double-stranded RNA adenosine deaminase (Bass, 1997. *Trends Biochem. Sci*. 22: 157-162). A simple explanation is that the primary transcripts from the *Nmy*[*sim2041*] and *nmy*[*sim1427*] region contain the full or partial inverted structure that forms a strong stem-loop structure which in turn stalls processing of reverse transcription. Small interfering RNAs are generated from the transcripts of *Nmy*[*sim2041*] but not from that of *nmy*[*sim1427*].
