# Supplementary material for: A sex-ratio Meiotic Drive System in Drosophila simulans. I: An Autosomal Suppressor
Source: PLoS Biol. 2007 Nov 6;5(11):e292. doi: 10.1371/journal.pbio.0050292 (PMC2062475; doi:10.1371/journal.pbio.0050292)
Supplement: Supplementary file 10 [file pbio.0050292.st001.doc]

**Table S1.** Sex Ratio Tested for Various Genotypes

| Crosses | n | Proportion of female (*k* ± SEM) |
| --- | --- | --- |
| 1. *SSR12* × *w; e* | 19 | 0.838 ± 0.009 ^a^ |
| 2. *SSR54* × *w; e* | 19 | 0.891 ± 0.032 ^a^ |
| 3. *SSR151* × *w; e* | 19 | 0.754 ± 0.018 ^a^ |
|  |  |  |
| 4. *NSR88* × *w; e* | 10 | 0.516 ± 0.015 |
| 5. *NSR113* × *w; e* | 10 | 0.491 ± 0.010 |
| 6. *NSR118* × *w; e* | 9 | 0.473 ± 0.014 |
|  |  |  |
| 7. *SSR12-2-7* × *w; e* | 43 | 0.895 ± 0.011 ^a^ |
| 8. *w; e* × *SSR12-2-7* (3 m × 10 f) | 10 | 0.519 ± 0.010 |
| 9. (*SSR12-2-7* × *w; e*) × *w; e* | 10 | 0.518 ± 0.017 |
| 10. (*w; e* × *SSR12-2-7*) × *w; e* | 8 | 0.494 ± 0.020 |
|  |  |  |
| 11. *SSR54-2-3* × *w; e* | 36 | 0.882 ± 0.015 ^a^ |
| 12. *w; e* × *SSR54-2-3* (3 m × 10 f) | 10 | 0.497 ± 0.019 |
|  |  |  |
| 13. *SSR151-2-10* × *w; e* | 37 | 0.945 ± 0.006 ^a^ |
| 14. *w; e* × *SSR151-2-10* (3 m × 10 f) | 10 | 0.537 ± 0.013 |
|  |  |  |
| 15. simB × *w; e* | 30 | 0.493 ± 0.012 |
| 16. *w; e* × simB (3 m × 10 f) | 10 | 0.499 ± 0.019 |
|  |  |  |
| 17. *SSR12-2-7* × *SSR54-2-3* (*en masse*) | 10 | 0.930 ± 0.013 |
| 18. (*SSR12-2-7* × *SSR54-2-3*) × *w; e* | 29 | 0.950 ± 0.006 |
| 19. *w; e* × (*SSR12-2-7* × *SSR54-2-3*) (3 m × 10 f) | 8 | 0.510 ± 0.017 |
|  |  |  |
| 20. *SSR54-2-3* × *SSR12-2-7* (*en masse*) | 10 | 0.897 ± 0.013 |
| 21. (*SSR54-2-3* × *SSR12-2-7*) × *w; e* | 27 | 0.939 ± 0.006 |
| 22. *w; e* × (*SSR54-2-3* × *SSR12-2-7*) (3 m × 10 f) | 10 | 0.527 ± 0.012 |
|  |  |  |
| 23. *SSR12-2-7* × *SSR151-2-10* (*en masse*) | 10 | 0.904 ± 0.021 |
| 24. (*SSR12-2-7* × *SSR151-2-10*) × *w; e* | 29 | 0.967 ± 0.006 |
| 25. *w; e* × (*SSR12-2-7* × *SSR151-2-10*) (3 m × 10 f) | 10 | 0.510 ± 0.012 |
|  |  |  |
| 26. (*SSR151-2-10* × *SSR12-2-7*) × *w; e* | 24 | 0.928 ± 0.007 |
| 27. *w; e* × (*SSR151-2-10* × *SSR12-2-7*) (3 m × 10 f) | 16 | 0.526 ± 0.007 |
|  |  |  |
| 28. *SSR54-2-3* × *SSR151-2-10* (*en masse*) | 10 | 0.942 ± 0.014 |
| 29. (*SSR54-2-3* × *SSR151-2-10*) × *w; e* | 29 | 0.947 ± 0.007 |
| 30. *w; e* × (*SSR54-2-3* × *SSR151-2-10*) (3 m × 10 f) | 10 | 0.518 ± 0.012 |
|  |  |  |
| 31. (*SSR151-2-10* × *SSR54-2-3*) × *w; e* | 29 | 0.909 ± 0.010 |
| 32. *w; e* × (*SSR151-2-10* × *SSR54-2-3*) (3 m × 10 f) | 10 | 0.536 ± 0.012 |
|  |  |  |
| 33. (*SSR12-2-7* × simB) × *w; e* | 30 | 0.490 ± 0.008 |
| 34. *w; e* × (*SSR12-2-7* × simB) (3 m × 10 f) | 10 | 0.502 ± 0.006 |
|  |  |  |
| 35. (simB × *SSR12-2-7*) × *w; e* | 29 | 0.504 ± 0.010 |
| 36. *w; e* × (simB × *SSR12-2-7*) (3 m × 10 f) | 10 | 0.502 ± 0.010 |
|  |  |  |
| 37. (*P*40 1-47 *Tmy* × *SSR12-2-7*) × *w; e* | 9 | 0.484 ± 0.014 ^b^ |
| 38. *w; e* × (*P*40 1-47 *Tmy* × *SSR12-2-7*) (3 m × 10 f) | 9 | 0.526 ± 0.008 ^b^ |
|  |  |  |
| 39. (*P*40 7-47 *tmy* × *SSR12-2-7*) × *w; e* | 8 | 0.504 ± 0.017 ^b^ |
| 40. *w; e* × (*P*40 7-47 *tmy* × *SSR12-2-7*) (3 m × 10 f) | 10 | 0.502 ± 0.011 ^b^ |
|  |  |  |

The cross is listed as male × female. All crosses were set up as 1 male × 3 *w; e* virgins if not otherwise indicated.

^a^ The sex ratios of two extracted lines (*SSR12-2-7* and *SSR151-2-10*) are significantly higher than that of the original *SSR* lines IG12 and IG151, respectively (*t*-test, *P* <0.001). However, this is not the case for *SSR54-2-3* as compared to IG54 (*P* = 0.794).

^b^ P40 7-47 *tmy* and P40 1-47 *Tmy* are heterozygous introgression lines where a *D. mauritiana* chromosomal fragment, covering the *too much yang* allele (*tmy*) or not (*Tmy*), respectively. The male genotype tested in cross 37 is *w*; *nt*; *P*40 1-47 *Tmy*/*SSR12-2-7* while that for cross 39 is *w*; *nt*; *P*40 7047 *tmy*/*SSR12-2-7*.
