# Supplementary material for: A sex-ratio Meiotic Drive System in Drosophila simulans. I: An Autosomal Suppressor
Source: PLoS Biol. 2007 Nov 6;5(11):e292. doi: 10.1371/journal.pbio.0050292 (PMC2062475; doi:10.1371/journal.pbio.0050292)
Supplement: Supplementary file 11 [file pbio.0050292.st002.doc]

**Table S2.** New ASO Markers Developed in this Study

| Name | Primer |  | Probe (wash °C ) | | |
| --- | --- | --- | --- | --- | --- |
| *CG3061.F* | GGAAACGTGTGAACTCGGAT |  | sim | ACAGGTCATAGTTCT | 45 |
| *CG3061.R* | TGTAGTATTGCCCGTGTCCA |  | mau | AGAACTACGACCTGT | 45 |
| *CG14362.F* | TGCTTTGCAGAAGTCTTGGA |  | sim | GTAATATAGGCTTCC | 43 |
| *CG14362.R* | GCTGCCCACCATATTGAACT |  | mau | GGAAGCCCATATTAC | 47 |
| *CG9322.F* | AAATCGATATGCTGGAACGC |  | sim | ACCCAATTTCGAAAT | 40 |
| *CG9322.R* | TCTTCCTAGCACGGCAAACT |  | mau | ATTTCAAAATTGGGC | 35 |
| *CG9654.F* | TGGATCTCTTCACCTCCACC |  | sim | CGCGCACGACTTTCT | 40 |
| *CG9654.R* | TTTGCGCAAGATCTTCATTG |  | mau | AGAAAATCGTACGCG | 35 |
| *CG10841.F* | GCGTATCGTCAGTTCTGCAA |  | sim | CATACAATTTAAGCACT | 25 |
| *CG10841.R* | TAGGTCCACTAAACGTCCCG |  | mau | CATTTAAATAAAGCACT | RT |
| *CG14368.F* | GCCAACAACATCCTTCTCGT |  | sim | ATCCACATCCTCGTC | 56 |
| *CG14368.R* | AAAACTGGCTTTGGTTGTGG |  | mau | GACGAGGCTGTGGAT | 56 |
| *K77.F* | gagaccgaagcctttgagtg |  | sim1 | CACTCAGAGAAAATA | 56 |
| *K77.R* | taggagcttgcggtctttgt |  | mau1 | TATTTTCCCTGAGTG | 56 |
| *K70.F2* | CATTTGCATGGCTTTCAGTG |  | sim | CTCTGGAGGCTTCAA | 45 |
| *K70.R2* | TCAAGGGATAACAAAAGGCG |  | mau | TTAAAGCCTCTAGAG | 45 |
| *K65.F* | cagctaacaagcgatgacca |  | sim | GGCAATACGGTTGGT | 53 |
| *K65.R* | actgacagaggcggaagaaa |  | mau | ACCAACCATATTGCC | 53 |
| *K60.F* | acacgacggggacaactaag |  | sim | AACATATACTCAATACTTC | 45 |
| *K60.R* | tttacttttcgatttgcggg |  | mau | GAAGTAATGAGTATACGTT | 45 |
| *K55.F* | gatacggtccctggagtcaa |  | sim | TGTGAGAACCCAAGG | 53 |
| *K55.R* | taggttgttttgcgggtagg |  | mau | CCTTGGGCTCTCACA | 53 |
| *CG14369.F* | ATAATAGCCAGGCGATGGTG |  | sim | TGCAGACTTCGCTGC | 50 |
| *CG14369.R* | ACAAGGATGTGGCACCAGTT |  | mau | TGCAGATTCCGCTGC | 50 |
| *K44.F* | gagcacaaagacaaggcaca |  | sim1 | GCAGGTGGGCGAAGA | 45 |
| *K44.R* | cgcaaccatgacaaatatcg |  | mau1 | GCAGGCAGTTGAAAA | 45 |

**Table S2** (cont.)

| Name | Primer |  | Probe (wash °C ) | | |
| --- | --- | --- | --- | --- | --- |
| *K40.F2* | cagcagcgtacataaagcca |  | sim1 | AACCAGCAGCACGTA | 47 |
| *K40.R2* | aaagctcagcatcagtcggt |  | mau1 | TACGTGCCGCTGGTT | 50 |
| *K35.F* | agcagctaccgaaatggcta |  | sim | ACTGGGCAGGATAAC | 56 |
| *K35.R* | aaaagcggcccaatagaaat |  | mau | GTTATCCAGCCCAGT | 56 |
| *K30.F* | ggcaacggtgaaattgtctt |  | sim | CGACGTGCTTTTGCG | 56 |
| *K30.R* | tttaaagcaagccccagcta |  | mau | CGCAAAAACACGTCG | 56 |
| *K25.F* | agccaaattgaaggtgatgg |  | sim | GTAGGTTTGTGGGGC | 56 |
| *K25.R* | gtggcattgtgtaatggctg |  | mau | GTGGGTTTGTGGAGC | 56 |
| *K20.F2* | GGCAGTCAGTTAAAGGCTCG |  | sim | GATTCCGATTCCGAT | 50 |
| *K20.R2* | TCAACCTATCGGATTCTCGG |  | mau | ATCGGACTCAGAATC | 50 |
| *CG31337.F* | CACAAGAACCAACTCGAGCA |  | sim | TGAGGCTAAACTTTTTA | 45 |
| *CG31337.R* | CTTGCGTCACTATGCGATGT |  | mau | TAAAAATTTAAGCCTCA | RT |
| *CG12538.F* | TGCTGTTCCAGTTGCTGTTC |  | sim | ACTAAAACGGGACCT | 45 |
| *CG12538.R* | GTCCGACGCTCTCAAAACTC |  | mau | AGGTCCCATTTTAGT | 45 |
| *CG14372.F* | GACGACGCCGTCTATATGGT |  | sim | ATTATCGTTGAAACC | 51 |
| *CG14372.R* | GATCCGGATGTTGTCGATCT |  | mau | GGTTTCAGCGATAAT | 50 |
| *CG9792.F* | GAAGAAGCGATGGAACTTCG |  | sim | TGTTCGTTCATATACAT | 40 |
| *CG9792.R* | CAGTCCGAGATTCATGAGCA |  | mau | ATGTATATAAACAAACG | 30 |
| *CG14375.F* | GGAATTGGCCAAGGGATAAT |  | sim | TCCGGTGCTCAGAGA | 48 |
| *CG14375.R* | GCCTACGGTCATGTGTGCTA |  | mau | TCTCTGAACACCGGA | 40 |
| *CG8784.F* | CAGCGACTGATGGCAGTTTA |  | sim | TTCTCAGCCAACCAC | 40 |
| *CG8784.R* | ATAGAGCTCCTGTTTCCGCA |  | mau | GTGGTTAGTTGAGAA | 35 |
| *CG8774.F* | GACTCTGTGGGTATTGCCGT |  | sim | GACTTTGTGCCCTGG | 45 |
| *CG8774.R* | GCTGCAGACAATCTGGAACA |  | mau | CCAAGGCACGAAGT | 40 |

All PCR’s use the same program: 94°C, 3’; (94°C, 15”; 55°C, 30”; 72°C, 30”) × 35, 72°C, 5’.
