# Supplementary material for: A sex-ratio Meiotic Drive System in Drosophila simulans. I: An Autosomal Suppressor
Source: PLoS Biol. 2007 Nov 6;5(11):e292. doi: 10.1371/journal.pbio.0050292 (PMC2062475; doi:10.1371/journal.pbio.0050292)
Supplement: Supplementary file 12 [file pbio.0050292.st003.doc]

**Table S3.** ASO Probes in the Last 7 kb Region of Fine Mapping

| Name | Probe (wash °C ) | |
| --- | --- | --- |
| *Nmy135_sim* | TTTTGGCATGCAGCA | 48 |
| *Nmy135_mau* | TGCTGCAAGCCAAAA | 48 |
| *Nmy529_sim* | GGGACTGCCGAAATG | 52 |
| *Nmy529_mau* | CATTTCGACAGTCCC | 48 |
| *Nmy2827_sim* | TTCGCATTCTTCTTC | 48 |
| *Nmy2827_mau* | TTCGCATCCTGGGAA | 35 |
| *Nmy3082_sim* | AACGTGCCATCATCA | 48 |
| *Nmy3082_mau* | AACGTGCTTAACTAG | 35 |
| *Nmy4357_sim* | GATCTCATATGGACTAA | 52 |
| *Nmy4357_mau* | TTAGTCCCTATGAGATC | 52 |
| *Nmy5585_sim* | GTGCAATTGAGCATAAT | 48 |
| *Nmy5585_mau* | GTGCATTTGAGATAGAC | 52 |
| *Nmy7123_sim* | AACGAACACATTCGG | 52 |
| *Nmy7123_mau* | CCGAATGCGTTCGTT | 52 |

Long PCR products of k50R30a and k44F30a from the 11 recombinants were hybridized to these probes.
