# Supplementary material for: A sex-ratio Meiotic Drive System in Drosophila simulans. I: An Autosomal Suppressor
Source: PLoS Biol. 2007 Nov 6;5(11):e292. doi: 10.1371/journal.pbio.0050292 (PMC2062475; doi:10.1371/journal.pbio.0050292)
Supplement: Supplementary file 13 [file pbio.0050292.st004.doc]

**Table S4.** Viability from Egg to Adult

| Strain | # Eggs | # Unhatched eggs | | # Hatched eggs | | Hatch rate L1/(L1+br) | Adults | | *k*  (m+f)/L1 | Survival  (m+f)/L1 |
| --- | --- | --- | --- | --- | --- | --- | --- | --- | --- | --- |
|  |  | White | Brown  (br) | # 1^st^ instar (L1) | Missing |  | Male  (m) | Female  (f) |  |  |
| *SSR12-2-7* | 1234 | 228 | 19 | 977 | 10 | 0.981 | 228 | 675 | 0.748 | 0.924 |
| *SSR54-2-3* | 959 | 184 | 19 | 746 | 10 | 0.975 | 114 | 552 | 0.829 | 0.893 |
| *SSR151-2-10* | 922 | 102 | 9 | 804 | 7 | 0.989 | 88 | 640 | 0.879 | 0.905 |
| simB | 918 | 162 | 10 | 737 | 9 | 0.987 | 323 | 314 | 0.493 | 0.864 |

White eggs were unfertilized while brown eggs were embryonic lethal. It is possible that some white eggs were fertilized but the fusion of the pronuclei had failed. This is clearly not the cause of *sex-ratio*, however, because the proportion of white eggs is similar between *SSR* lines and simB control.
